# Commentary: Genome-Based Taxonomic Classification of the Phylum *Actinobacteria*

**DOI:** 10.3389/fmicb.2019.00206

**Published:** 2019-02-22

**Authors:** Radhey S. Gupta

**Affiliations:** Department of Biochemistry and Biomedical Sciences, McMaster University, Hamilton, ON, Canada

**Keywords:** family *Mycobacteriaceae* and the order *Mycobacteriales*, genus *Mycobacterium*, order *Corynebacteriales*, division of the family *Mycobacteriaceae* into five genera, prokaryotic taxonomy, Priority of taxonomic names

Nouioui et al. (2018) have recently reported comprehensive phylogeny and taxonomy of members from the phylum Actinobacteria. Based on genomic-scale analyses, this study makes proposals for many new taxa of Actinobacteria. A significant portion of this study concerns the classification of species from the order *Corynebacteriales*. This study and another study by Goodfellow and Jones (2012) indicate the order *Corynebacteriales* as containing several families including *Mycobacteriaceae*. However, *Mycobacteriaceae* is a part of the validly published order *Mycobacteriales* as proposed by Janke (1924) and included in the Approved Lists of bacterial names (Skerman et al., 1980). In contrast, the order *Corynebacteriales* was proposed in 2012 (Goodfellow and Jones, 2012) and validly published in 2015 (Oren and Garrity, 2015). Thus, according to Rules 23a and 24a of the International Code of Nomenclature of Prokaryotes (Parker et al., 2015), the order name *Mycobacteriales* Janke 1924 has priority over the name *Corynebacteriales* Goodfellow and Jones 2015 and all of the families which are part of the order *Corynebacteriales* should be embedded within the order *Mycobacteriales*. To correct this anomaly, an emended description of the order *Mycobacteriales*, based partially on the description of the *Corynebacteriales* by Goodfellow and Jones (2012) and Nouioui et al. (2018), is provided below.

The genus *Mycobacterium* previously consisted of 188 named species (Parte, 2014). Our recent comprehensive phylogenomics and comparative analyses of 150 mycobacterial genomes robustly demonstrated the grouping of these species into five distinct clades (Gupta et al., 2018). Besides their distinct separation in genomic-scale phylogenetic trees and in average amino acid identity matrices, the members of these clades were also reliably demarcated based on multiple molecular synapomorphies uniquely shared by members from each clade (Figure 1A). The compelling evidence from these studies formed the basis for our division of the genus *Mycobacterium* into five genera. In this classification, the genus name *Mycobacterium* is retained for the clade containing all major human and animal pathogens (e.g., *M. tuberculosis, M. leprae, M. bovis*, etc.), whereas species from other clades, harboring mainly non-pathogenic species, are transferred into four new genera viz. *Mycolicibacterium, Mycolicibacter, Mycolicibacillus*, and *Mycobacteroides* (Figure 1A).

**Figure 1:**
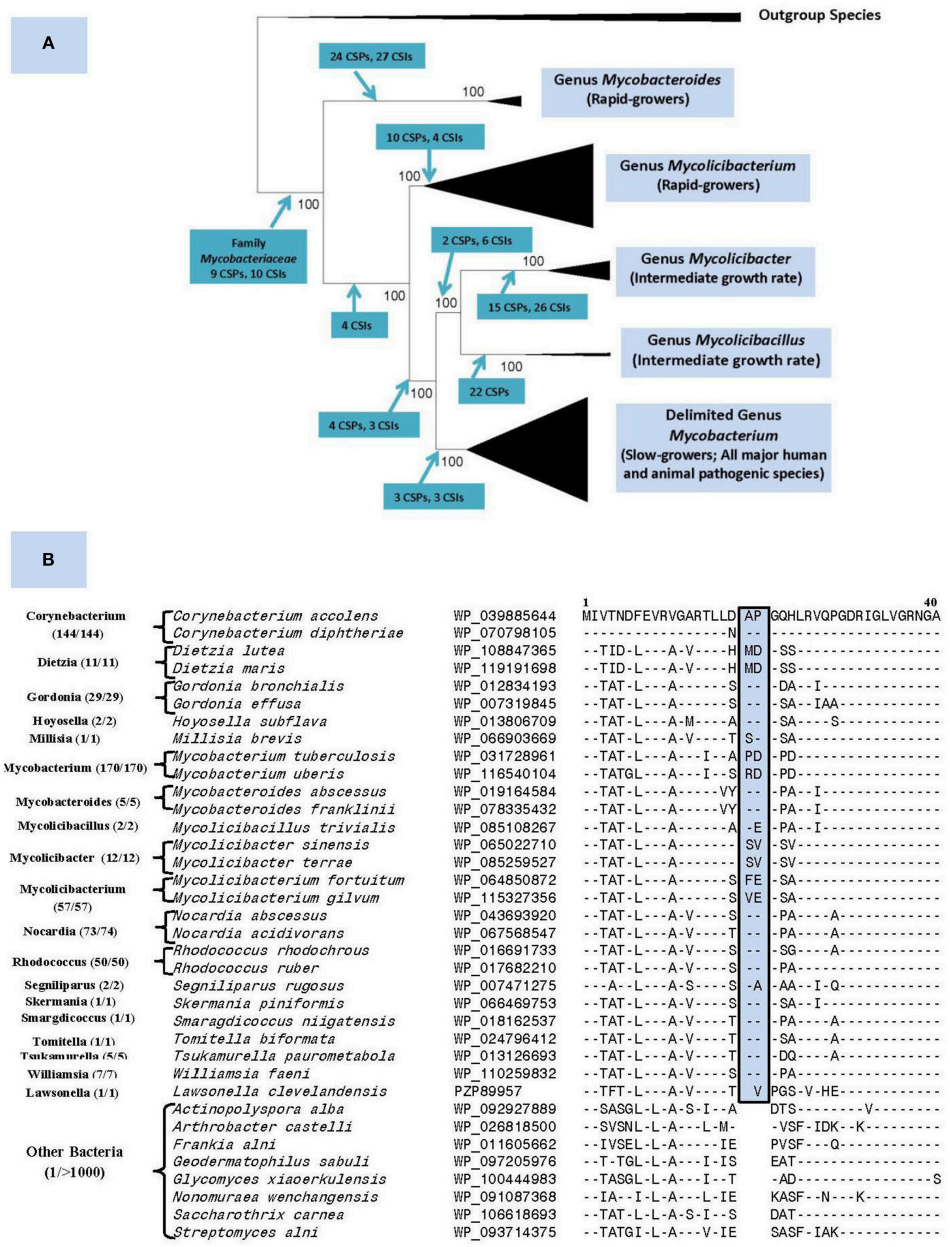
**(A)** A summary diagram showing the five main clades within the family *Mycobacteriaceae*, now recognized as distinct genera, seen in different phylogenetic trees. The molecular synapomorphies consisting of conserved signature indels (CSIs) and conserved signature proteins (CSPs), which distinguish different clades are marked on the nodes. The members of these clades/genera also differ in their growth rates. **(B)** Partial sequence alignment of the ABC-F family ATP-binding cassette domain-containing protein showing a 2 aa insert in a conserved region that is commonly and uniquely present in different members of the order *Mycobacteriales*. The dashes (–) in the alignment show identity with the amino acids shown on the top line. The numbers with the genus name refer to different unique strains for which sequences were examined. The sequence information is shown for only 1–2 representative species from different genera that are part of this order and very few outgroup species. However, this CSI is not found in other Actinobacteria or other examined bacteria (see Figure S1) with the possible exception of *Kroppenstedtia sanguinis* (a Firmicute). This CSI as well as a number of other CSIs and CSPs for this order were described in our earlier work (Gao and Gupta, 2012) based on limited sequence information and most of them are still distinctive characteristics of this order.

Nouioui et al. (2018) commend our work for identifying molecular synapomorphies for the five mycobacterial clades and their phylogenetic analysis and those by Tortoli et al. (2017), also support the existence of these clades. However, while not questioning any aspects of our results, Nouioui et al. (2018) question our division of the genus *Mycobacterium* into five genera for two reasons. First, they indicate that our identification of several synapomorphies for all mycobacteria makes further division of this group into multiple genera arbitrary. However, molecular synapomorphies can exist at different phylogenetic/taxonomic levels ranging from phylum to the genus levels (Gupta, 2016). We have previously identified synapomorphies for the phylum Actinobacteria as well as several classes/orders within this phylum (Gao and Gupta, 2012). For the class *Coriobacteriia*, synapomorphies were identified for the entire class along with its two orders and three families (Gupta et al., 2013). In fact, we have also described a number of CSIs and CSPs specific for the order *Corynebacteriales* (now emended order *Mycobacteriales*) (Gao and Gupta, 2012). Updated sequence information show that all three described CSIs and two CSPs are uniquely shared by different *Mycobacteriales* species with only isolated exceptions (see Figure 1B). Therefore, the existence of molecular synapomorphies for a higher taxonomic clade should not preclude its further division into lower level taxa, whose monophyly is strongly supported by independent means.

Nouioui et al. (2018) are also critical of our proposal on the grounds that some *Mycobacteriales* genera viz. *Rhodococcus, Gordonia, Nocardia*, and *Corynebacterium* are more divergent than *Mycobacterium*. However, in comparison to these genera, interspecies relationships within the genus *Mycobacterium* are now much better understood, because of our comprehensive genomic analyses. Additionally, *Mycobacterium* species also differ in their growth rates and this distinction is also supported by our classification scheme. Another important rationale for dividing the genus *Mycobacterium* is that it contains some of the most important human pathogens, including *M. tuberculosis*, the causative agent of tuberculosis (TB). For developing improved means for detection and treatment of TB, it is crucial to understand how the TB-causing group of bacteria differ from other related bacteria (Gupta, 2018; Gupta et al., 2018). In this context, our classification scheme, which separates all major human and animal pathogenic species (retained within the genus *Mycobacterium*) from other mycobacteria, constitutes an important step. With this division, attention can be focused on the unique genetic and molecular characteristics of the clinically important group of mycobacteria (Gupta, 2018; Gupta et al., 2018). Thus, we emphasize here that the reservations expressed by Nouioui et al. (2018) of our proposed division of the genus *Mycobacterium* are not justified.

## Emended Description of the Order *Mycobacteriales* Janke 1924 (Approved Lists 1980)

The order is comprised of aerobic or facultatively anaerobic, chemoorganotrophic species exhibiting Gram-positive or acid-fast staining response. Most members are catalase- positive and form a branched substrate mycelium that fragments into coccoid- to rod-shaped elements or present as branched filaments, cocci, or as pleomorphic forms. Some strains form aerial hyphae. The wall peptidoglycan contains *meso*-diaminopimelic acid and is of the A1g type. Arabinose and galactose are major wall sugars. Fatty acid profiles are rich in saturated and unsaturated components and usually contain tuberculostearic acid. Mycolic acids are important constituents of the cell envelopes of most members. Members of this order form a monophyletic clade in phylogenetic trees based on 16S rRNA and large datasets of protein sequences. In addition, the following conserved signature indels (CSIs), viz. 2 aa insert in ABC-F family ATP-binding protein (Uup), 1 aa insert in chromosome partitioning protein ParB, and 1 aa deletion in alpha-ketoglutarate decarboxylase (KGD) and the homologs of the two conserved signature proteins (CSPs) with accession numbers NP_301197.1 and NP_301204.1 are also primarily found in the members of this order (Gao and Gupta, 2012). The order contains the families *Corynebacteriaceae, Dietziaceae, Gordoniaceae, Lawsonellaceae, Mycobacteriaceae, Nocardiaceae, Segniliparaceae*, and *Tsukamurellaceae*.

Type genus: *Mycobacterium* Lehmann and Neumann, 1896 (Approved Lists 1980) emend. Gupta et al. 2018

## Author Contributions

The author confirms being the sole contributor of this work and has approved it for publication.

### Conflict of Interest Statement

The author declares that the research was conducted in the absence of any commercial or financial relationships that could be construed as a potential conflict of interest. The reviewer VS and handling editor declared their shared affiliation at the time of the review.
