# Supplementary material for: Commentary: Genome-Based Taxonomic Classification of the Phylum Actinobacteria
Source: Front Microbiol. 2019 Feb 22;10:206. doi: 10.3389/fmicb.2019.00206 (PMC6395429; doi:10.3389/fmicb.2019.00206)
Supplement: Supplementary file 1 [file Image_1.pdf]

Figure S1

Detailed information regarding the species distribution of the 2 aa consert insert in the ABC-F family ATP-binding cassette domain-containing protein shown in Figure 1B, which is specifically found in members of the order *Mycobacteriales*. The dashes (-) in this alignment show identity with the amino acids shown on the top line.

Corynebacterium (144/144)

Species	Accession	1		44
Corynebacterium accolens	WP_039885644	MIVTNDFEVRVGARTLLD	AP	GQHLRVQPGDRIGLVGRNGAGKTT
Corynebacterium afermentans	WP_063936733	------L-----------	--	------------------------
Corynebacterium ammoniagenes	WP_003845875	----Q-L----------E	--	--Q-----------I---------
Corynebacterium amycolatum	WP_005510820	----H-L----------T	--	-EQ---------------------
Corynebacterium appendicis	WP_076598584	------L----------H	--	-------A----------------
Corynebacterium aquilae	WP_075726230	---V-------------N	--	------------------------
Corynebacterium argentoratense	WP_020976291	------L----------N	--	--L---------------------
Corynebacterium atypicum	WP_038605405	----Q-L-----S----T	S-	-EQ---------------------
Corynebacterium aurimucosum	WP_010186726	------------------	--	------------------------
Corynebacterium auriscanis	WP_035113173	------L----------N	--	--L---------------------
Corynebacterium bouchesdurhone	WP_066525440	------L-----------	--	------------------------
Corynebacterium bovis	WP_010271064	----Q-L-----------	--	--L---------------------
Corynebacterium callunae	WP_015651348	------L-----------	--	--L---------------------
Corynebacterium camporealensis	WP_046453390	------------S-----	--	-------A----------------
Corynebacterium capitovis	WP_018017030	----Q-L----------T	--	-------A----------------
Corynebacterium casei	WP_025387563	----Q-L----------E	--	--Q-----------I---------
Corynebacterium caspium	WP_018339790	----R-L----------T	--	-EQ---------------------
Corynebacterium choanis	WP_123927919	---AS-L-----S---VQ	FS	-D----------------------
Corynebacterium ciconiae	WP_026161453	------L----------H	--	------------------------
Corynebacterium coyleae	WP_092102603	------L-----------	--	------------------------
Corynebacterium crudilactis	WP_066565780	------L-----------	--	--L---------------------
Corynebacterium cystitidis	WP_092255305	------L----------E	--	--Q---------------------
Corynebacterium deserti	WP_053544964	------L----------N	--	--L---------------------
Corynebacterium diphtheriae	WP_070798105	-----------------N	--	------------------------
Corynebacterium doosanense	WP_018021687	--A-K-L----------N	--	-A-----A---V------------
Corynebacterium durum	WP_040359867	------C----------H	--	------------------------
Corynebacterium efficiens	WP_006767679	------L----------T	--	------------------------
Corynebacterium epidermidicani	WP_047240257	---SE-L----------N	--	------------------------
Corynebacterium falsenii	WP_025402695	------L----------N	--	------------------------
Corynebacterium flavescens	WP_075729837	------------------	--	--L---------------------
Corynebacterium fournierii	WP_085958001	------L-----------	--	-----------V------------
Corynebacterium frankenforsten	WP_075663794	----H-L----------T	--	-DQ---------------------
Corynebacterium freiburgense	WP_027012321	----E------------F	--	------------------------
Corynebacterium freneyi	WP_035124069	----H-L-----------	--	-EM---------------------
Corynebacterium genitalium	WP_005290086	------L----------N	--	-------A----------------
Corynebacterium geronticis	WP_123934079	------L----------E	--	------------------------
Corynebacterium glaucum	WP_095659996	------L-----------	--	------------------------
Corynebacterium glucuronolytic	SMB81106	V---Q-L----------T	--	------------------------
Corynebacterium glutamicum	WP_096455989	------L----------N	--	--L---------------------
Corynebacterium glyciniphilum	WP_038548586	----Q-L----------N	--	--Q---------------------
Corynebacterium hadale	WP_095548177	------L----------S	--	-------S----------------
Corynebacterium halotolerans	WP_015400932	------L----------T	--	-------A---V------------
Corynebacterium heidelbergense	WP_113631111	----Q------------N	--	------------------------
Corynebacterium humireducens	WP_040085835	------L----------T	--	------------------------
Corynebacterium ihumii	WP_034998947	------L-----------	--	------------------------
Corynebacterium imitans	WP_038590694	------L----------N	--	------------------------
Corynebacterium jeddahense	WP_042404754	------L-----------	--	------------------------
Corynebacterium jeikeium	WP_111712100	------L----------N	--	------------------------
Corynebacterium kroppenstedtii	PZR04194	--T-E-LD---------T	--	-H-----A----------------
Corynebacterium kutscheri	WP_046441250	-----------------Y	--	------------------------
Corynebacterium lactis	WP_053413308	----H-L----------T	-D	-E----------------------
Corynebacterium lipophiloflavu	WP_006840284	----H-L----------T	--	------------------------
Corynebacterium lowii	WP_055178529	------L----------T	--	-------A----------------
Corynebacterium lubricantis	WP_018295544	------L----------E	--	--Q---------------------
Corynebacterium macginleyi	WP_121910694	-----------------A	--	------------------------
Corynebacterium marinum	WP_042621463	------L----------T	--	--L---------------------
Corynebacterium maris	WP_020934804	------L----------T	--	-----------V------------
Corynebacterium massiliense	WP_022862015	------L----------E	--	-H-----H------I---------
Corynebacterium mastitidis	WP_026166153	------L----------T	--	-------A----------------
Corynebacterium matruchotii	WP_040432014	-----------------T	--	K-Q---------------------
Corynebacterium minutissimum	WP_039675286	------------------	--	------------------------
Corynebacterium mustelae	WP_047262102	-----------------Q	--	--Q---------------------

Corynebacterium (144/144)

Corynebacterium mycetoides	WP_092148485	----Q-L----------N	--	-----------V------------
Corynebacterium nuruki	WP_010118502	----Q-L-----------	--	--L---------------------
Corynebacterium oculi	WP_055121870	------L----------T	--	-------A----------------
Corynebacterium otitidis	WP_004600578	--A-S-L-I---S----T	--	-GQ--I------------------
Corynebacterium phocae	WP_075734042	-----------------E	--	--M---------------------
Corynebacterium phoceense	WP_068801786	------------------	--	------------------------
Corynebacterium pilosum	WP_018581990	------L----------E	--	------------------------
Corynebacterium pollutisoli	WP_085549976	------L----------T	--	------------------------
Corynebacterium propinquum	WP_018119566	---SQ-L---A------H	--	--------------I---------
Corynebacterium provencense	WP_066589716	----Q-L----------N	--	--L---------------------
Corynebacterium pseudodiphther	WP_024272920	---SQ-L---A------H	--	--------------I---------
Corynebacterium pseudogenitali	WP_040425243	------------------	--	--------------I---------
Corynebacterium pseudopelargi	WP_123960190	------L----------H	--	------------------------
Corynebacterium pseudotubercul	WP_014558703	----------A------N	--	------------------------
Corynebacterium pyruviciproduc	WP_040426814	----Q-L----------T	--	------------------------
Corynebacterium renale	WP_111726070	----K-M----------T	--	-EQ---------------------
Corynebacterium resistens	WP_013888430	------L----------N	--	--M---------------------
Corynebacterium riegelii	WP_052204222	------L-----------	--	------------------------
Corynebacterium simulans	WP_062035079	------------------	--	------------------------
Corynebacterium singulare	WP_042530781	------------------	--	------------------------
Corynebacterium sputi	WP_027020524	----H-L-----------	--	-EL----E----------------
Corynebacterium sp. 13CS0277	WP_106015028	---V-------------H	-E	------------------------
Corynebacterium sp. BCW_4722	WP_075466821	------L-----------	--	------------------------
Corynebacterium sp. CCUG 69366	WP_123047776	---S--L----------T	--	------H-----------------
Corynebacterium sp. CMW7794	WP_066513327	------------------	--	------------------------
Corynebacterium sp. CNJ-954	WP_075815997	----Q-L----------N	--	--Q---------------------
Corynebacterium sp. DNF00584	KXB52198	V-----------------	--	------------------------
Corynebacterium sp. HFH0082	WP_016422758	----H-L----------T	--	-EQ---------------------
Corynebacterium sp. HMSC034E11	WP_070820263	------L-----------	--	------------------------
Corynebacterium sp. HMSC036D02	WP_070818355	------------------	--	------------------------
Corynebacterium sp. HMSC036E10	WP_070828985	------L-----------	--	------------------------
Corynebacterium sp. HMSC04H06	WP_070768021	------L----------N	--	--------------I---------
Corynebacterium sp. HMSC055D05	WP_070570352	------L-----------	--	------------------------
Corynebacterium sp. HMSC059E07	WP_070684981	------------------	--	------------------------
Corynebacterium sp. HMSC05E07	WP_070536479	------------------	--	------------------------
Corynebacterium sp. HMSC062A03	WP_070643505	------------------	--	------------------------
Corynebacterium sp. HMSC063A05	WP_070586015	----H-L----------T	--	-EQ---------------------
Corynebacterium sp. HMSC067D03	WP_070615032	------L-----------	--	------------------------
Corynebacterium sp. HMSC06G04	WP_070498436	------------------	--	------------------------
Corynebacterium sp. HMSC071B10	WP_070762712	------L----------H	--	------------------------
Corynebacterium sp. HMSC072A02	WP_070564491	------------------	--	------------------------
Corynebacterium sp. HMSC073H12	WP_070832032	----H-L----------T	--	-EQ---------------------
Corynebacterium sp. HMSC074A01	WP_070789319	------L----------N	--	------------------------
Corynebacterium sp. HMSC074C01	WP_070744093	------------------	--	------------------------
Corynebacterium sp. HMSC074C03	WP_070852229	----H-L----------T	--	-EQ---------------------
Corynebacterium sp. HMSC074E01	WP_070528368	------------------	--	------------------------
Corynebacterium sp. HMSC076C10	WP_070643468	----H-L----------T	--	-EQ---------------------
Corynebacterium sp. HMSC078H07	WP_070671193	------------------	--	------------------------
Corynebacterium sp. HMSC08C04	WP_070455618	------------------	--	------------------------
Corynebacterium sp. HMSC11D10	WP_070451443	------L-----------	--	------------------------
Corynebacterium sp. HMSC11E11	WP_070522307	----H-L-----------	--	-EM---------------------
Corynebacterium sp. HMSC11H10	WP_070432243	----H-L----------T	--	-EQ---------------------
Corynebacterium sp. HMSC22B11	WP_070758905	----K-L----------T	--	-E----------A-----------
Corynebacterium sp. HMSC27B11	WP_070761416	----K-L----------T	--	-E----------A-----------
Corynebacterium sp. HMSC28B08	WP_070433624	------L----------N	--	--L---------------------
Corynebacterium sp. HMSC29G08	WP_070462239	------L----------T	--	------------------------
Corynebacterium sp. HMSC30G07	WP_070494930	------L----------T	--	------------------------
Corynebacterium sp. J010B-136	WP_105324992	----Q-L----------E	--	--Q-----------I---------
Corynebacterium sp. JB4	WP_098072737	----Q-L----------E	--	--Q-----S-----I---------
Corynebacterium sp. KPL1855	ERS56326	V-----------------	--	------------------------
Corynebacterium sp. KPL1995	ERS38486	V--SQ-L---A------H	--	--------------I---------
Corynebacterium sp. NML 120412	WP_095545756	------L----------S	--	------------------------
Corynebacterium sp. NML130628	WP_071566280	----T-L----------E	--	------------------------
Corynebacterium sp. NML140438	WP_071568272	----T-L----------E	--	------------------------
Corynebacterium sphenisci	WP_075693843	----K-L---A-------	--	-EQ---------------------
Corynebacterium spheniscorum	WP_092286181	------L----------T	--	------------------------
Corynebacterium stationis	WP_066794045	----Q-L----------E	--	--Q-----------I---------
Corynebacterium striatum	WP_114976249	------------------	--	------------------------
Corynebacterium terpenotabidum	WP_020441145	----Q-L----------N	--	------------------------
Corynebacterium testudinoris	WP_047253115	------L----------T	--	------------------------
Corynebacterium timonense	WP_019194610	------------------	--	------------------------
Corynebacterium tuberculostear	WP_005328391	------------------	--	--------------I---------
Corynebacterium tuscaniense	WP_034663384	------L----------N	--	------------------------
Corynebacterium ulcerans	WP_102240991	-----------------N	--	------------------------

	Corynebacterium ulceribovis	WP_018024008	----Q-L----------S	--	-E---------------------
	Corynebacterium urealyticum	WP_012360263	----K-L----------T	--	-E----------A----------
	Corynebacterium ureicelerivora	WP_038611655	----H-L----------Q	--	-----------------------
	Corynebacterium urinapleomorph	WP_087117949	------L----------N	--	-------A---------------
	Corynebacterium uterequi	WP_047259601	------L----------E	--	-A---------------------
	Corynebacterium variabile	WP_041630566	----Q-L----------N	--	--L--------------------
	Corynebacterium vitaeruminis	WP_025252867	-----------------N	--	-----------------------
	Corynebacterium xerosis	WP_102211888	----H-L-----------	--	-EM--------------------
**Dietzia (11/11)**	Dietzia alimentaria	WP_010541733	--TID-L---A-V----H	MD	-SS----R---------------
	Dietzia cinnamea	WP_007626465	--TID-L---A-V----Q	MD	-SS--------------------
	Dietzia lutea	WP_108847365	--TID-L---A-V----H	MD	-SS--------------------
	Dietzia maris	WP_119191698	--TID-L---A-V----H	MD	-SS--------------------
	Dietzia natronolimnaea	WP_095716931	--TID-L---A-V----H	MD	-SS--------------------
	Dietzia psychralcaliphila	WP_107747589	--TID-L---A-V----H	MD	-SS--------------------
	Dietzia sp. 111N12-1	WP_067716291	--TID-L---A-V----H	MD	-SS--------------------
	Dietzia sp. JS16-p6b	WP_107756487	--TID-L---A-V----H	MD	-SS--------------------
	Dietzia sp. UCD-THP	WP_017836796	--TID-L---A-V----H	MD	-SS--------------------
	Dietzia sp. WMMA184	WP_096905457	--TID-L---A-V----H	MD	-SS--------------------
	Dietzia timorensis	WP_067471116	--TID-L---A-V----H	MD	--T--------------------
**Gordonia (29/29)**	Gordonia aichiensis	WP_005173400	--TAT-L---A------S	--	-SA--I-----------------
	Gordonia amarae	WP_005181056	--TAT-L---A------S	--	-SA--I-----------------
	Gordonia amicalis	WP_006436247	--TAT-L---A------S	--	-DA--I-----------------
	Gordonia araii	WP_007323223	--TAT-L---A------S	--	-AA--IG----------------
	Gordonia bronchialis	WP_012834193	--TAT-L---A------S	--	-DA--I-----------------
	Gordonia effusa	WP_007319845	--TAT-L---A------S	--	-SA--IAA---------------
	Gordonia hirsuta	WP_024332857	--TAT-L---A------S	--	-DA--IA----------------
	Gordonia hydrophobica	WP_066171925	--TAT-L---A------S	--	-DA---A----------------
	Gordonia iterans	WP_105942370	--TAT-L---A------S	--	-DA--IA----------------
	Gordonia kroppenstedtii	WP_026303588	--TAT-L---A-V----S	--	-PA--I-----------------
	Gordonia lacunae	WP_086535459	--TAT-L---A------S	--	-DA--I-----------------
	Gordonia malaquae	WP_008377842	--TAT-L---A------S	--	-DQ--I-----------------
	Gordonia namibiensis	WP_006866274	--TAT-L---A------S	--	-DA--I-----------------
	Gordonia neofelifaecis	WP_009678212	--TAT-L---A------S	--	-D---I-----------------
	Gordonia otitidis	WP_007241142	--TAT-L---A------S	--	-SAM-I-----------------
	Gordonia paraffinivorans	WP_006899496	--TAT-L---A------S	--	-DA--I-----------------
	Gordonia phthalatica	WP_062395236	--TAT-L---A------A	--	-DA---A----------------
	Gordonia polyisoprenivorans	WP_014359975	--TAT-L---A------S	--	-SA--I-----------------
	Gordonia rhizosphera	WP_006338846	--TAT-L---A------S	--	-SA--I-----------------
	Gordonia rubripertincta	WP_005194929	--TAT-L---A------S	--	-DA--I-----------------
	Gordonia rubripertincta	WP_119032364	--TAT-L---A------S	--	-DA--I-----------------
	Gordonia shandongensis	WP_026917888	--TAT-L---A------S	--	-DA---A----------------
	Gordonia soli	WP_007621147	--TAT-L---A------S	--	-SA--IA----------------
	Gordonia sp. 1D	WP_096273972	--TAT-L---A------S	--	-DA--I-----------------
	Gordonia sp. KTR9	WP_014926684	--TAT-L---A------S	--	-DA--I-----------------
	Gordonia sp. MMS17-SY073	WP_124707226	--TAT-L---A------S	--	-SA--I-----------------
	Gordonia sp. RS15-1S	WP_123925178	--TAT-L---A------S	--	-SA--I-----------------
	Gordonia terrae NBRC 100016	GAB45071	V-TAT-L---A------S	--	-DA--I-----------------
	Gordonia westfalica	WP_074848991	--TAT-L---A------S	--	-DA--I-----------------
**Hoyosella (2/2)**	Hoyosella altamirensis	WP_064438809	--TAT-L---A-M----A	--	-SA--------------------
	Hoyosella subflava	WP_013806709	--TAT-L---A-M----A	--	-SA----S---------------
**Millisia (1/1)**	Millisia brevis	WP_066903669	--TAT-L---A-V----T	S-	-SA--------------------
**Mycobacterium (170/170)**	Mycobacterium ahvazicum	SOX53548	V-TAT-L---A---I--S	PD	-PD--I-----------------
	Mycobacterium alsense	WP_067318394	--TAT-L---A---I--S	PD	-PD--------------------
	Mycobacterium angelicum	WP_083114101	--TAT-L---A---I--S	PD	-PD--I-----------------
	Mycobacterium aquaticum	WP_083160896	--TAT-L---A------S	IE	-SA--I-----------------
	Mycobacterium arosiense	WP_083064033	--TAT-L---A---I--S	PD	-PD--------------------
	Mycobacterium asiaticum	WP_036355708	--TATGL---A---I--S	PD	-PD--------------------
	Mycobacterium avium	WP_009977557	--TAT-L---A---I--S	PD	-PD--------------------
	Mycobacterium bohemicum	WP_085179979	--TAT-L---A---I--S	PD	-PD--------------------
	Mycobacterium branderi	WP_083134605	--TAT-L---A------S	VD	-AA--I-----------------
	Mycobacterium canettii	WP_014000787	--TAT-L---A---I--A	PD	-PD--------------------
	Mycobacterium chimaera	WP_074021427	--TAT-M---A---I--S	PD	-PD--------------------
	Mycobacterium colombiense	WP_007774262	--TAT-L---A---I--S	PD	-PD--------------------
	Mycobacterium conspicuum	WP_085232765	--TAT-L---A---I--S	PD	-PD--------------------
	Mycobacterium decipiens	WP_085325129	--TAT-L---A---I--S	PD	-PD--------------------
	Mycobacterium dioxanotrophicus	WP_087077741	--TAT-L---A------S	IE	-SA--I-----------------
	Mycobacterium europaeum	WP_090423223	--TAT-L---A---I--S	PD	-PD--------------------
	Mycobacterium florentinum	WP_085221815	--TAT-L---A---I--S	PD	-PD--I-----------------
	Mycobacterium fragae	WP_085196731	--TAT-L---A------S	-D	-PA--------------------
	Mycobacterium gastri	WP_036414818	--TAT-L---A---I--S	PE	-PD--------------------
	Mycobacterium genavense	WP_025735830	--TAT-L---A---I--S	PD	-PD--I-----------------
	Mycobacterium gordonae	WP_065132661	--TAT-L---A---I--S	PD	-PD--------------------
	Mycobacterium grossiae	WP_070354975	--TAT-L---A------S	IE	-AA--------------------
	Mycobacterium haemophilum	WP_047313848	--TAT-L---A---I--S	PD	-PD--------------------

Mycobacterium (170/170)

Mycobacterium heckeshornense	WP_048892697	--TATGL---A------S	VD	-SG---------------------
Mycobacterium heidelbergense	WP_083075231	--TAT-L---A---I--S	PD	-PD---------------------
Mycobacterium interjectum	WP_085202930	--TAT-L---A---I--S	PD	-PD---------------------
Mycobacterium intermedium	WP_069421710	--TAT-L---A---I--S	PD	-PD---------------------
Mycobacterium intracellulare	WP_014380445	--TAT-L---A---I--S	PD	-PD---------------------
Mycobacterium kansasii	WP_023373345	--TAT-L---A---I--S	PE	-PD---------------------
Mycobacterium kubicae	WP_068031853	--TAT-L---A---I--S	PD	-PD---------------------
Mycobacterium kyorinense	WP_045375409	--TAT-L---A------S	TD	-AA--I------------------
Mycobacterium lacus	WP_085163020	--TATGL---A---I--S	PD	-PD---------------------
Mycobacterium lentiflavum	WP_090601591	--TAT-L---A---I--S	PD	-PD--I------------------
Mycobacterium leprae	WP_010908557	--TAT-L---A---I--S	SD	-PD---------------------
Mycobacterium lepraemurium	ATA28337	V-TAT-L---A---I--S	PD	-PD---------------------
Mycobacterium lepromatosis	WP_045843415	--TAT-L---A---I--S	PD	-PD---------------------
Mycobacterium liflandii	WP_015356083	--TAT-L---A---I--S	PD	-PD---------------------
Mycobacterium malmoense	WP_065442133	--TAT-L---A---I--S	PD	-PD---------------------
Mycobacterium mantenii	WP_067836158	--TAT-L---A---I--S	PD	-PD--I------------------
Mycobacterium marinum	WP_020728610	--TAT-L---A---I--S	PD	-PD---------------------
Mycobacterium marseillense	WP_067168223	--TAT-L---A---I--S	PD	-PD--I------------------
Mycobacterium montefiorense	WP_108922725	--TAT-L---A---I--S	PD	-PD--I------------------
Mycobacterium nebraskense	WP_046185053	--TAT-L---A---I--S	PD	-PD---------------------
Mycobacterium neumannii	WP_094294758	--TAT-L---A------S	TD	-TA---------------------
Mycobacterium noviomagense	WP_083085945	--TAT-L---A---I--S	-D	-PA----S----------------
Mycobacterium palustre	WP_085080735	--TAT-L---A---I--A	PD	-PD---------------------
Mycobacterium paraense	WP_085244872	--TAT-L---A---I--S	PD	-PD---------------------
Mycobacterium paraffinicum	WP_073872520	--TAT-L---A---I--S	PD	-PD---------------------
Mycobacterium paraseoulense	WP_083173813	--TAT-L---A---I--S	PD	-PD---------------------
Mycobacterium parmense	WP_085269625	--TAT-L---A---I--S	PD	-PD---------------------
Mycobacterium persicum	WP_075546076	--TAT-L---A---I--S	PE	-PD---------------------
Mycobacterium riyadhense	WP_085252196	--TAT-L---A---I--S	PD	-PD---------------------
Mycobacterium saskatchewanense	WP_085257074	--TAT-L---A---I--S	PD	-PD---------------------
Mycobacterium scrofulaceum	WP_083178801	--TAT-L---A---I--S	PD	-PD---------------------
Mycobacterium sherrisii	WP_069401196	--TATGL---A---I--S	PD	-PD--I------------------
Mycobacterium shigaense	WP_096439455	--TAT-L---A---I--S	PD	-PD--I------------------
Mycobacterium shimoidei	WP_113965070	--TAT-L---A------S	-D	-PA---------------------
Mycobacterium shinjukuense	WP_083049183	--TAT-L---A---I--S	PG	-PD---------------------
Mycobacterium simiae	WP_044511428	--TATGL---A---I--S	PD	-PD--I------------------
Mycobacterium sp. 1081908.1	WP_067014729	--TAT-L---A---I--S	PD	-PD---------------------
Mycobacterium sp. 1100029.7	WP_066823056	--TAT-L---A---I--S	PE	-PD--I------------------
Mycobacterium sp. 1164966.3	WP_067377755	--TAT-L---A---I--S	PD	-PA---------------------
Mycobacterium sp. 1164985.4	WP_067291207	--TAT-L---A------S	TD	-TA---------------------
Mycobacterium sp. 1165196.3	WP_067184196	--TAT-L---A---I--S	PD	-PD---------------------
Mycobacterium sp. 1245111.1	WP_067333477	--TAT-L---A------Y	-D	-PA--I------------------
Mycobacterium sp. 1245805.9	WP_067160717	--TAT-L---A---I--S	PD	-PD---------------------
Mycobacterium sp. 1245852.3	WP_067140326	--TAT-L---A---I--S	PD	-PD---------------------
Mycobacterium sp. 1274756.6	WP_066851142	--TAT-L---A------A	TD	-AA--I------------------
Mycobacterium sp. 1274761.0	WP_066987209	--TAT-L---A------S	-D	-TA---------------------
Mycobacterium sp. 141	WP_019973468	--TAT-L---A------S	IE	-SA--I------------------
Mycobacterium sp. 1465703.0	WP_066988351	--TAT-L---A---I--S	PD	-PD---------------------
Mycobacterium sp. 155	WP_018600206	--TAT-L---A------S	ID	-SA---------------------
Mycobacterium sp. 1554424.7	WP_066929502	--TAT-L---A---I--S	SD	-PD---------------------
Mycobacterium sp. 3519A	WP_101949868	--TAT-L---A------S	TE	-TA---------------------
Mycobacterium sp. 4858	WP_102417846	--TAT-L---A---I--S	PD	-PD---------------------
Mycobacterium sp. 852002-10029	WP_067251738	--TAT-L---A---I--S	PD	-PD--I------------------
Mycobacterium sp. 852002-30065	WP_067202365	--TAT-L---A---I--S	PD	-PD--I------------------
Mycobacterium sp. 852002-40037	WP_067097805	--TAT-L---A---I--S	PD	-PD---------------------
Mycobacterium sp. 852002-50816	WP_066967062	--TAT-L---A---I--A	PD	-PD--I------------------
Mycobacterium sp. 852002-51057	WP_067114020	--TAT-L---A---I--S	PD	-PD---------------------
Mycobacterium sp. 852002-51152	WP_066850387	--TAT-L---A------S	TD	-TA---------------------
Mycobacterium sp. 852002-51163	WP_067240694	--TAT-L---A---I--S	PD	-PA--I------------------
Mycobacterium sp. 852002-51961	WP_067081434	--TAT-L---A------S	TD	-TA---------------------
Mycobacterium sp. 852002-51971	WP_067132749	--TAT-L---A---I--S	PD	-PD---------------------
Mycobacterium sp. 852013-50091	WP_064944251	--TAT-L---A------S	IE	-SA--I------------------
Mycobacterium sp. 852013-51886	WP_066831023	--TAT-L---A------S	VE	-SA---------------------
Mycobacterium sp. 852014-50255	WP_067741550	--TAT-L---A---I--S	PD	-PD--I------------------
Mycobacterium sp. 852014-52144	WP_068201545	--TAT-L---A------S	TD	-TA---------------------
Mycobacterium sp. 852014-52450	WP_067789243	--TAT-L---A---I--S	PD	-PD---------------------
Mycobacterium sp. AB215	WP_077081445	--TAT-L---A---I--S	PD	-PD---------------------
Mycobacterium sp. AB308	WP_077098784	--TAT-L---A---I--S	PD	-PD---------------------
Mycobacterium sp. AB57	WP_077089714	--TATGL---A---I--S	PD	-PD--I------------------
Mycobacterium sp. ACS1612	WP_067810779	--TAT-L---A------S	TD	-TA---------------------
Mycobacterium sp. ACS4054	WP_067898383	--TAT-L---A---I--S	PD	-PD---------------------
Mycobacterium sp. ACS4331	WP_068256093	--TAT-L---A------L	-D	-TT--I------------------
Mycobacterium sp. AT1	WP_079922879	--TAT-L---A------A	IE	-SA--I------------------
Mycobacterium sp. CECT 8778	WP_099037561	--TAT-L---A------S	TE	-AA---------------------

Mycobacterium (170/170)

Mycobacterium sp. CECT 8779	WP_099022234	--TAT-L---A------L	-E	-PA---------------------
Mycobacterium sp. D16R12	WP_078295651	--TAT-L---A-----VY	--	-PA--I------------------
Mycobacterium sp. D16R18	WP_078359450	--TAT-L---A-----VY	--	-PA--I------------------
Mycobacterium sp. E136	WP_068293683	--TAT-L---A------S	TD	-TA---------------------
Mycobacterium sp. E1715	WP_068259393	--TAT-L---A---I--S	PE	-PD---------------------
Mycobacterium sp. E1747	WP_068077041	--TAT-L---A---I--S	PD	-PD---------------------
Mycobacterium sp. E2327	WP_068108363	--TAT-L---A---I--S	PD	-PD---------------------
Mycobacterium sp. E2462	WP_068281907	--TAT-L---A---I--S	PD	-PD--I------------------
Mycobacterium sp. E2479	WP_067934972	--TAT-L---A---I--S	PD	-PD---------------------
Mycobacterium sp. E2497	WP_068093215	--TAT-L---A---I--S	PD	-PD---------------------
Mycobacterium sp. E2733	WP_068041895	--TAT-L---A---I--S	PD	-PD---------------------
Mycobacterium sp. E3198	WP_068232675	--TAT-L---A---I--S	PD	-PD---------------------
Mycobacterium sp. E3247	WP_067925576	--TAT-L---A---I--S	PD	-PD---------------------
Mycobacterium sp. E3251	WP_068011878	--TAT-L---A---I--S	PD	-PD---------------------
Mycobacterium sp. E3298	WP_067780598	--TAT-L---A---I--S	PD	-PD---------------------
Mycobacterium sp. E3305	WP_067754062	--TAT-L---A---I--S	PE	-PD---------------------
Mycobacterium sp. E342	WP_068056158	--TAT-L---A---I--S	PD	-PD---------------------
Mycobacterium sp. E735	WP_067890760	--TAT-L---A---I--S	PE	-PD---------------------
Mycobacterium sp. E740	WP_068143723	--TAT-L---A------S	TD	-TA---------------------
Mycobacterium sp. E796	WP_068127109	--TAT-L---A---I--S	PD	-PD---------------------
Mycobacterium sp. E802	WP_067763605	--TAT-L---A------S	FE	-SA---------------------
Mycobacterium sp. ENV421	WP_102807743	--TAT-L---A------L	-E	-PA--I------------------
Mycobacterium sp. EPG1	WP_104860888	--TAT-L---A------S	IE	-SA---------------------
Mycobacterium sp. EPa45	WP_047330359	--TAT-L---A------L	-E	-PA--I------------------
Mycobacterium sp. GA-1199	WP_064348388	--TAT-L---A------S	TD	-TA---------------------
Mycobacterium sp. GA-1285	WP_064423924	--TAT-L---A------S	TD	-TA---------------------
Mycobacterium sp. GA-1841	WP_076195535	--TAT-L---A------S	FE	-SA---------------------
Mycobacterium sp. GA-2829	WP_059092804	--TAT-L---A------S	VE	-AA---------------------
Mycobacterium sp. H4Y	WP_008257989	--TAT-L---A---I--S	PD	-PD---------------------
Mycobacterium sp. IS-1264	WP_076062900	--TAT-L---A---I--S	PD	-PD---------------------
Mycobacterium sp. IS-1496	WP_059159437	--TAT-L---A------Y	TE	-TT---------------------
Mycobacterium sp. IS-1590	WP_064406108	--TAT-L---A------S	TD	-TA---------------------
Mycobacterium sp. IS-1742	WP_059095542	--TAT-L---A------Y	TE	-TT---------------------
Mycobacterium sp. IS-2888	WP_076115747	--TAT-L---A---I--S	PD	-PD---------------------
Mycobacterium sp. IS-3022	WP_059164051	--TAT-L---A------S	TD	-TA---------------------
Mycobacterium sp. IS-836	WP_076124863	--TAM-L---A---I--S	PD	-PD---------------------
Mycobacterium sp. ITM-2016-003	WP_105344139	--TAT-L---A------S	-E	-PA---------------------
Mycobacterium sp. ITM-2017-009	PRC43239	--TAT-L---A------S	IE	-SA---------------------
Mycobacterium sp. JS623	WP_015306799	--TAT-L---A------S	TD	-AA---------------------
Mycobacterium sp. M26	WP_059019830	--TAT-L---A------L	-E	-PA---------------------
Mycobacterium sp. MFM001	WP_116375786	--TAT-L---A------S	TD	-AA--I------------------
Mycobacterium sp. MS1601	WP_083740297	--TAT-L---A------A	-E	-PA---------------------
Mycobacterium sp. NAZ190054	WP_067963749	--TAT-L---A------S	IE	-SA---------------------
Mycobacterium sp. NS-7484	WP_076240614	--TAT-L---A------S	FE	-SA---------------------
Mycobacterium sp. PYR10	WP_099961414	--TAT-L---A------S	VE	-AA---------------------
Mycobacterium sp. PYR15	WP_096312845	--TAT-L---A------L	-E	-PA--I------------------
Mycobacterium sp. QGD 101	WP_102141000	--TAT-L---A------S	TD	-TA---------------------
Mycobacterium sp. Root135	WP_056548094	--TAT-L---A------A	IE	-SA--I------------------
Mycobacterium sp. Root265	WP_057165121	--TAT-L---A------S	IE	-SA--I------------------
Mycobacterium sp. SP-6446	WP_076247281	--TAT-L---A---I--S	PD	-PD---------------------
Mycobacterium sp. ST-F2	WP_073696584	--TAT-L---A------A	FE	-SA--I------------------
Mycobacterium sp. Soil538	WP_057146423	--TAT-L---A------A	IE	-AA---------------------
Mycobacterium sp. UM_Kg1	WP_046319253	--TAT-L---A-------	-V	-SV---------------------
Mycobacterium sp. UM_Kg17	WP_046296838	--TATGL---A-------	SA	DSV---------------------
Mycobacterium sp. UM_NZ2	WP_046282994	--TATGL---A-------	SV	DSV---------------------
Mycobacterium sp. UM_WWY	WP_029371249	--TAT-L---A------S	IE	-SA--I------------------
Mycobacterium sp. UNC267MFSha1	WP_090358002	--TAT-L---A------S	IE	-AA--I------------------
Mycobacterium sp. UNC280MFTsu5	WP_043396406	--TAT-L---A------A	FE	-SA--I------------------
Mycobacterium sp. URHB0044	WP_029115508	--TAT-L---A------S	VE	-AA--I------------------
Mycobacterium sp. URHD0025	WP_029107585	--TAT-L---A------S	IE	-SA---------------------
Mycobacterium sp. WY10	WP_071949876	--TAT-L---A------L	-E	-PA--I------------------
Mycobacterium sp. YC-RL4	WP_067997770	--TAT-L---A------S	IE	-SA--I------------------
Mycobacterium sp. YR782	WP_108056177	--TAT-L---A------L	-E	-PA---------------------
Mycobacterium sp. djl-10	WP_068919816	--TAT-L---A------A	-E	-PA---------------------
Mycobacterium sp. shizuoka-1	WP_099251411	--TAT-L---A------L	-E	-PA---------------------
Mycobacterium syngnathidarum	WP_070943675	--TAT-L---A------S	IE	-SA---------------------
Mycobacterium szulgai	WP_085671920	--TAT-L---A---I--S	PD	-PD--I------------------
Mycobacterium talmoniae	PQM47465	--TAT-L---A------S	-A	-PA---------------------
Mycobacterium triplex	WP_036468673	--TAT-L---A---I--S	PD	-PD--I------------------
Mycobacterium tuberculosis	WP_031728961	--TAT-L---A---I--A	PD	-PD---------------------
Mycobacterium uberis	WP_116540104	--TATGL---A---I--S	RD	-PD---------------------
Mycobacterium ulcerans	WP_011739623	--TAT-V---A---I--S	PD	-PD---------------------
Mycobacterium xenopi	WP_085198444	--TAT-L---A---I--S	-D	-PT---------------------

Genus	Species	Accession	Sequence	Boxed	Sequence
**Mycobacteroides (5/5)**	Mycobacteroides abscessus	WP_019164584	--TAT-L---A-----VY	--	-PA--I------------------
	Mycobacteroides franklinii	WP_078335432	--TAT-L---A-----VY	--	-PA--I------------------
	Mycobacteroides immunogenum	WP_043075615	--TAT-L---A-----VY	--	-PA--I------------------
	Mycobacteroides salmoniphilum	WP_078327051	--TAT-L---A-----VY	--	-PA--I------------------
	Mycobacteroides saopaulense	WP_070910298	--TAT-L---A-----VY	--	-PA--I------------------
**Mycolicibacillus (2/2)**	Mycolicibacillus koreensis	WP_085301732	--TAT-L---A-------	-T	-AA--I------------------
	Mycolicibacillus trivialis	WP_085108267	--TAT-L---A------A	-E	-PA--I------------------
**Mycolicibacter (12/12)**	Mycolicibacter algericus	WP_083039531	--TATGL---A-------	SV	DSV---------------------
	Mycolicibacter arupensis	WP_046189419	--TATGL---A-------	SV	DSV---------------------
	Mycolicibacter engbaekii	WP_085128860	--TATGL---A-------	SV	DSV---------------------
	Mycolicibacter heraklionensis	WP_076050103	--TATGL---A-------	SV	DSV---------------------
	Mycolicibacter hiberniae	WP_085134611	--TAT-L---A-------	-V	-SV---------------------
	Mycolicibacter icosiumassilien	WP_067973133	--TAT-L---A------A	-E	-PA--I------------------
	Mycolicibacter longobardus	WP_085263840	--TATGL---A-------	SV	DSV--I------------------
	Mycolicibacter minnesotensis	WP_083024666	--TAT-L---A------A	-E	-PA--I------------------
	Mycolicibacter nonchromogenicu	WP_085137128	--TATGL---A-------	SV	DSV---------------------
	Mycolicibacter senuensis	WP_085087007	--TATGL---A-------	SV	DSV---------------------
	Mycolicibacter sinensis	WP_065022710	--TAT-L---A-------	SV	-SV---------------------
	Mycolicibacter terrae	WP_085259527	--TAT-L---A-------	SV	-SV---------------------
**Mycolicibacterium (57/57)**	Mycolicibacterium acapulense	WP_064419825	--TAT-L---A------S	TD	-TA---------------------
	Mycolicibacterium agri	WP_097941446	--TAT-L---A------S	TD	-TA---------------------
	Mycolicibacterium aichiense	WP_115319800	--TAT-L---A------L	-E	-PA--I------------------
	Mycolicibacterium aromaticivor	WP_036337839	--TAT-L---A------L	-E	-PA--I------------------
	Mycolicibacterium aurum	WP_048633926	--TAT-L---A------S	VE	-SA---------------------
	Mycolicibacterium bacteremicum	WP_083054539	--TAT-L---A------S	IE	-AA--I------------------
	Mycolicibacterium boenickei	WP_077739327	--TAT-L---A------S	ID	-SA---------------------
	Mycolicibacterium brisbanense	WP_062831858	--TAT-L---A------S	IE	-SA--I------------------
	Mycolicibacterium brumae	WP_090585112	--TAT-L---A------A	-E	-PA---------------------
	Mycolicibacterium canariasense	WP_062655504	--TAT-L---A------S	IE	-AA---------------------
	Mycolicibacterium celeriflavum	WP_067223658	--TAT-L---A------S	TD	-TA---------------------
	Mycolicibacterium chubuense	WP_014815713	--TAT-L---A------S	ID	-SA---------------------
	Mycolicibacterium confluentis	WP_085154768	--TAT-L---A------L	-D	-TT--I------------------
	Mycolicibacterium diernhoferi	WP_073854766	--TAT-L---A------S	IE	-SA--I------------------
	Mycolicibacterium doricum	WP_085191534	--TAT-L---A------Y	TE	-TT---------------------
	Mycolicibacterium duvalii	WP_098001261	--TAT-L---A------S	VE	-SA---------------------
	Mycolicibacterium elephantis	WP_083043522	--TAS-L---A------S	TD	-SV---------------------
	Mycolicibacterium fallax	WP_085098540	--TAT-L---A------A	-E	-PA--I------------------
	Mycolicibacterium flavescens	WP_069412177	--TAT-L---A------S	TD	-TA---------------------
	Mycolicibacterium fortuitum	WP_064850872	--TAT-L---A------S	FE	-SA---------------------
	Mycolicibacterium gilvum	WP_115327356	--TAT-L---A------S	VE	-SA---------------------
	Mycolicibacterium goodii	WP_049746922	--TAT-L---A------S	ID	-AA---------------------
	Mycolicibacterium hassiacum	WP_005627370	--TAT-L---A------S	TN	-TA---------------------
	Mycolicibacterium holsaticum	WP_069407224	--TAT-L---A------S	TD	-SA---------------------
	Mycolicibacterium houstonense	WP_066897232	--TAT-L---A------S	ID	-SA---------------------
	Mycolicibacterium insubricum	WP_083030479	--TAT-L---A------A	-D	-PA---------------------
	Mycolicibacterium iranicum	WP_064284999	--TAT-L---A------S	TE	-SA---------------------
	Mycolicibacterium komanii	WP_090278695	--TAT-L---A------S	TD	-TA---------------------
	Mycolicibacterium litorale	WP_078019634	--TAT-L---A------H	-E	-PA---------------------
	Mycolicibacterium llatzerense	WP_043987420	--TAT-L---A------A	FE	-SA--I------------------
	Mycolicibacterium malmesburyen	WP_090341745	--TAT-L---A------S	TD	-SA---------------------
	Mycolicibacterium monacense	WP_064917056	--TAT-L---A------S	ID	-AA---------------------
	Mycolicibacterium moriokaense	WP_114738344	--TAT-L---A------A	TD	-TA---------------------
	Mycolicibacterium mucogenicum	WP_061000875	--TAT-L---A------A	FE	-SA--I------------------
	Mycolicibacterium neoaurum	WP_030135638	--TAT-L---A------S	IE	-SA--I------------------
	Mycolicibacterium neworleansen	WP_090511787	--TAT-L---A------S	IE	-SA---------------------
	Mycolicibacterium novocastrens	WP_064416263	--TAT-L---A------S	TD	-TA---------------------
	Mycolicibacterium parafortuitu	WP_083143366	--TAT-L---A------S	IE	-SA---------------------
	Mycolicibacterium peregrinum	WP_055113747	--TAT-L---A------S	FE	-SA---------------------
	Mycolicibacterium phlei	WP_003885901	--TAT-L---A------S	TN	-TA---------------------
	Mycolicibacterium porcinum	WP_065462190	--TAT-L---A------S	IE	-SA---------------------
	Mycolicibacterium rhodesiae	WP_014213508	--TAT-L---A------S	TE	-AA---------------------
	Mycolicibacterium rufum	KGI68116	--TAT-L---A------E	ID	-AA---------------------
	Mycolicibacterium rutilum	WP_083409001	--TAT-L---A------S	TD	-TA---------------------
	Mycolicibacterium septicum	WP_044518303	--TAT-L---A------S	FE	-SA---------------------
	Mycolicibacterium setense	WP_039379494	--TAT-L---A------S	IE	-SA---------------------
	Mycolicibacterium smegmatis	WP_003894527	--TAT-L---A------L	IE	-SA---------------------
	Mycolicibacterium sp. CECT 878	WP_123026393	--TAT-L---A------S	TD	-TA---------------------
	Mycolicibacterium sp. GF69	WP_112553591	--TAS-L---A------S	TD	-TA---------------------
	Mycolicibacterium sphagni	WP_094478172	--TAT-L---A------L	-E	-PA---------------------
	Mycolicibacterium tokaiense	WP_115281346	--TAT-L---A------A	-E	-PA---------------------
	Mycolicibacterium tusciae	WP_083123397	--TAT-L---A------S	-E	-AA---------------------
	Mycolicibacterium vaccae	WP_003932035	--TAT-L---A------S	IE	-SA---------------------

	Mycolicibacterium vanbaalenii	ABM13549	V-TAT-L---A------S	IE	-SA---------------------
	Mycolicibacterium vulneris	WP_085289413	--TAT-L---A---I--S	PD	-PD---------------------
	Mycolicibacterium wolinskyi	WP_067845325	--TAT-L---A------S	TE	-AA---------------------
Nocardia (73/74)	Nocardia abscessus	WP_043693920	--TAT-L---A-V----S	--	-PA----A----------------
	Nocardia acidivorans	WP_067568547	--TAT-L---A-V----T	--	-PA----A----------------
	Nocardia africana	WP_062964551	--TAT-L---A-I----T	--	-SA----A----------------
	Nocardia alba	WP_067452770	--TAT-L---A-V----S	--	-PA----S----------------
	Nocardia altamirensis	WP_069167141	--TAT-L---A-V----S	--	-PA----A----------------
	Nocardia amamiensis	WP_067466027	--TAT-L---A-V----S	--	-PA----A----------------
	Nocardia amikacinitolerans	WP_067780845	--TAT-L---A-V----S	--	-PA----A----------------
	Nocardia anaemiae	WP_062991893	--TAT-L---A-V----S	--	-PA----A----------------
	Nocardia araoensis	WP_039798697	--TAT-L---A-V----S	--	-PA----A----------------
	Nocardia arizonensis	WP_054812996	--TAT-L---A-V----S	--	-SA----A----------------
	Nocardia arthritidis	WP_063053191	--TAT-L---A-V----S	--	-PA----A----------------
	Nocardia asiatica	WP_043727121	--TAT-L---A-V----S	--	-PA----A----------------
	Nocardia beijingensis	WP_067801780	--TAT-L---A-V----S	--	-PA----A----------------
	Nocardia brasiliensis	WP_014986329	--TAT-L---A-V----S	--	-PA----S----------------
	Nocardia brevicatena	WP_040836203	--TAT-L---A-I----S	--	-PA----T----------------
	Nocardia caishijiensis	WP_067981104	--TAT-L---A-V----S	--	-PA----S----------------
	Nocardia carnea	WP_033246205	--TAT-L---A-V----S	--	-PA----A----------------
	Nocardia cerradoensis	WP_039778843	--TAT-L---A-I----T	--	-SA----A----------------
	Nocardia concava	WP_040815309	--TAT-L---A-V----T	--	-SG----A----------------
	Nocardia crassostreae	WP_067536815	--TAT-L---A-V----T	--	-SA----A----------------
	Nocardia cyriacigeorgica	WP_014350845	--TAT-L---A-V----S	--	-PA----A----------------
	Nocardia donostiensis	WP_077115216	--TAT-L---A-V----S	--	-PA----A----------------
	Nocardia farcinica	WP_068971572	--TAT-L---A------A	--	-DA--I------------------
	Nocardia flavorosea	WP_062971141	--TAT-L---A-V----S	--	-PA----A----------------
	Nocardia fluminea	WP_101468688	--TAT-L---A-V----S	--	-PA----S----------------
	Nocardia fusca	WP_063128946	--TAT-L---A-V----S	--	-PA----A----------------
	Nocardia grenadensis	WP_063034326	--TAT-L---A-V----S	--	-PA----A----------------
	Nocardia harenae	WP_067649709	--TAT-L---A-V----T	--	-SA----N----------------
	Nocardia higoensis	WP_040797005	--TAT-L---A-V----S	--	-PA----A----------------
	Nocardia inohanensis	WP_067814871	--TAT-L---A-V----T	--	-SA----A----------------
	Nocardia jejuensis	WP_067701306	--TAT-L---A-V----T	--	-SA----S----------------
	Nocardia jiangxiensis	WP_040824719	--TAT-L---A-I----T	--	-SA----S----------------
	Nocardia jinanensis	WP_058856259	--TAT-L---A-V----S	--	-PA----A----------------
	Nocardia lijiangensis	WP_067848499	--TAT-L---A-V----S	--	-PA----A----------------
	Nocardia mexicana	WP_068031921	--TAT-L---A-I----T	--	-AA----S----------------
	Nocardia mikamii	WP_062996166	--TAT-L---A-I----T	--	-SA----A----------------
	Nocardia miyunensis	WP_067683696	--TAT-L---A-I----T	--	-SA----S----------------
	Nocardia neocaledoniensis	WP_110035467	--TAT-L---A-V----S	--	-PA----S----------------
	Nocardia niigatensis	WP_040862941	--TAT-L---A-V----T	--	-SA----A----------------
	Nocardia niwae	WP_063017843	--TAT-L---A-V----S	--	-PA----A----------------
	Nocardia nova	WP_025350344	--TAT-L---A-I----T	--	-SA----A----------------
	Nocardia otitidiscaviarum	WP_029924995	--TAT-L---A-V----T	--	-SA----A----------------
	Nocardia paucivorans	WP_040790537	--TAT-L---A-I----S	--	-PA----A----------------
	Nocardia pneumoniae	WP_040779974	--TAT-L---A-V----S	--	-PA----A----------------
	Nocardia pseudobrasiliensis	WP_062515006	--TAT-L---A-I----T	--	-SA----A----------------
	Nocardia puris	WP_067513623	--TAT-L---A-V----S	--	-PA----A----------------
	Nocardia rhamnosiphila	WP_030520515	--TAT-L---A-V----S	--	-PA----A----------------
	Nocardia seriolae	WP_063058080	--TAT-L---A-V----S	--	-PA----A----------------
	Nocardia sp. 348MFTsu5.1	WP_028463511	--TAT-L---A-V----S	--	-PA---------------------
	Nocardia sp. BMG111209	WP_019926751	--TAT-L---A-V----T	--	-SA----A----------------
	Nocardia sp. BMG51109	WP_024805260	--TAT-L---A-I----T	--	-SA----A----------------
	Nocardia sp. CFHS0054	WP_120744222	--TAT-L---A-V----T	--	-SA----A----------------
	Nocardia sp. CNY236	WP_028476764	--TAT-L---A-I----S	--	-PA----S----------------
	Nocardia sp. FDAARGOS_372	WP_098083988	--TAT-L---A-V----S	--	-PA----A----------------
	Nocardia sp. NEAU-LL90	WP_122190000	--TAT-L---A-I----T	--	-SA----A----------------
	Nocardia sp. NRRL WC-3656	WP_030514545	--TAT-L---A-I----T	--	-SA----A----------------
	Nocardia sp. SYSU K10002	WP_109526973	--TAT-L---A-V----S	--	-PA----A----------------
	Nocardia sp. Y48	WP_071928852	--TAT-L---A-V----S	--	-PM----S----------------
	Nocardia sp. YIM PH 21724	WP_120045076	--TAT-L---A-V----S	--	-PA----A----------------
	Nocardia speluncae	WP_068036134	--TAT-L---A-V----S	--	-PA----A----------------
	Nocardia suismassiliense	WP_107660659	--TAT-L---A-V----S	--	-PA----S----------------
	Nocardia takedensis	WP_040708209	--TAT-L---A-V----S	--	-SA----A----------------
	Nocardia tenerifensis	WP_040736163	--TAT-L---A-V----S	--	-PA----S----------------
	Nocardia terpenica	WP_067588423	--TAT-L---A-I----T	--	-PA----A----------------
	Nocardia testacea	WP_039825422	--TAT-L---A-V----S	--	-PA----A----------------
	Nocardia thailandica	WP_043653281	--TAT-L---A-V----S	--	-PA----A----------------
	Nocardia transvalensis	WP_040750825	--TAT-L---A-I----T	--	-SA----A----------------
	Nocardia uniformis	WP_067520228	--TAT-L---A-V----T	--	-PA----S----------------
	Nocardia vaccinii	WP_067893387	--TAT-L---A-I----T	--	-SA----S----------------
	Nocardia vermiculata	WP_067880266	--TAT-L---A-I----T	--	-SA----A----------------
	Nocardia veterana	WP_040719777	--TAT-L---A-I----T	--	-SA----A----------------

Nocardia (73/74)	Nocardia xishanensis	WP_068064909	--TAT-L---A-V----S	--	-PA----A----------------
	Nocardia yamanashiensis	WP_067722743	--TAT-L---A-V----T	--	-SA----A----------------
	Nocardia shimofusensis	WP_067854880	--TAT-L---A-V----S	--	-PA----A----------------
	Nocardia sienata fii	WP_072808496	--TAT-L---A-V----S	--	-SA----A----------------
	Nocardia sp. NRRL S-836	WP_053736508	--TAT-M-L-A-S-I-VS		-AN---------------------
Rhodococcus (50/50)	Rhodococcus biphenylivorans	WP_019289301	--TAT-L---A-V----T	--	-SG----A----------------
	Rhodococcus coprophilus	WP_072698293	--TAT-L---A-V----S	--	-SA----A----------------
	Rhodococcus corynebacterioides	WP_068152036	--TAT-L---A-V----S	--	-PA--I-A----------------
	Rhodococcus defluvii	WP_031937474	--TAT-L---A-V----S	--	-PA----A----------------
	Rhodococcus erythropolis	WP_019748419	--TAT-L---A-V----T	--	-PS--I------------------
	Rhodococcus fascians	WP_037142315	--TAT-L---A-V----S	--	-PA----A----------------
	Rhodococcus gordoniae	WP_064064887	--TAT-L---A-V----T	--	-SG----A----------------
	Rhodococcus hoagii	WP_013415964	--TAT-L---A-V----S	--	-SA----A----------------
	Rhodococcus jostii	WP_073370673	--TAT-L---A-V----S	--	-PA---------------------
	Rhodococcus kroppenstedtii	WP_068363289	--TAT-L---A-V----S	--	-PA----A----------------
	Rhodococcus kunmingensis	WP_068271248	--TAT-L---A-V----S	--	-SA----A----------------
	Rhodococcus kyotonensis	WP_068424222	--TAT-L---A-V----S	--	-PA----A----------------
	Rhodococcus maanshanensis	SEL69356	L-TAT-L---A-V----S	--	-PA----A----------------
	Rhodococcus marinonascens	WP_072688730	--TAT-L---A-V----S	--	-PA---------------------
	Rhodococcus opacus	WP_005255032	--TAT-L---A-V----S	--	-PA---------------------
	Rhodococcus phenolicus	WP_068157179	--TAT-L---A-V----S	--	-PA----A----------------
	Rhodococcus pyridinivorans	WP_024100823	--TAT-L---A-V----T	--	-SG----A----------------
	Rhodococcus qingshengii	WP_083057180	--TAT-L---A-V----T	--	-PS--I------------------
	Rhodococcus rhodnii	WP_040774763	--TAT-L---A-V----T	--	-SA--I-A----------------
	Rhodococcus rhodochrous	WP_016691733	--TAT-L---A-V----S	--	-SG----A----------------
	Rhodococcus ruber	WP_017682210	--TAT-L---A-V----S	--	-PA---------------------
	Rhodococcus sp. 06-235-1A	WP_094674202	--TAT-L---A-V----S	TE	-PA----A----------------
	Rhodococcus sp. 1139	WP_070162277	--TAT-L---A-V----T	--	-PS--I------------------
	Rhodococcus sp. 1163	WP_084348849	--TAT-L---A-V----S	TQ	-PA----G----------------
	Rhodococcus sp. 1168	WP_088943139	--TAT-L---A-V----S	TQ	-PA----G----------------
	Rhodococcus sp. 14-2483-1-2	WP_094741965	--TAT-L---A-V----S	IE	-PA----A----------------
	Rhodococcus sp. 15-649-1-2	WP_094711029	--TAT-L---A-V----S	--	-PA----A----------------
	Rhodococcus sp. 2G	WP_071934903	--TAT-L---A-V----S	--	-SG----A----------------
	Rhodococcus sp. AW25M09	WP_008716484	--TAT-L---A-V----S	TE	-PA----A----------------
	Rhodococcus sp. BUPNP1	WP_088898528	--TAT-L---A-V----S	--	-SG----A----------------
	Rhodococcus sp. ENV425	WP_102799751	--TAT-L---A-V----S	--	-PA---------------------
	Rhodococcus sp. EPR-157	WP_068368395	--TAT-L---A-V----M	--	-PA----A----------------
	Rhodococcus sp. Eu-32	WP_114329523	--TAT-L---A-V----S	--	-PA----A----------------
	Rhodococcus sp. HS-D2	WP_064256289	--TAT-L---A-V----T	--	-SG----A----------------
	Rhodococcus sp. Leaf278	WP_057478701	--TAT-L---A-V----S	TE	-PA----A----------------
	Rhodococcus sp. NCIMB 12038	WP_087561339	--TAT-L---A-V----S	--	-PA---------------------
	Rhodococcus sp. OK269	WP_115960130	--TAT-L---A-V----S	--	-PA----A----------------
	Rhodococcus sp. OK302	WP_094272831	--TAT-L---A-V----T	--	-PS--I------------------
	Rhodococcus sp. OK519	WP_107985040	--TAT-L---A-V----S	--	-PA----A----------------
	Rhodococcus sp. P1Y	WP_121114913	--TAT-L---A-V----S	--	-PA--I-G----------------
	Rhodococcus sp. PBTS 1	WP_068101181	--TAT-L---A-V----S	--	-PA----A----------------
	Rhodococcus sp. RD6.2	WP_050064233	--T-T-L---A-T----T	-S	-PA----A----------------
	Rhodococcus sp. RD6.2	WP_050065940	--TAT-L---A-V----S	--	-SA----A----------------
	Rhodococcus sp. S2-17	WP_109335128	--TAT-L---A-V----S	--	-PA---------------------
	Rhodococcus sp. WMMA185	WP_070378513	--TAT-L---A-V----S	--	-PA---------------------
	Rhodococcus triatomae	WP_072738214	--TAT-L---A-V----S	--	-PA--I------------------
	Rhodococcus triatomae	WP_007535406	--TAT-L---A-V----S	--	-SA----A----------------
	Rhodococcus wratislaviensis	WP_112300788	--TAT-L---A-V----S	--	-PA---------------------
	Rhodococcus yunnanensis	WP_072801864	--TAT-L---A-V----S	--	-PA----A----------------
	Rhodococcus zop	WP_033087058	--TAT-L---A-V----T	--	-SA----S----------------
Segniliparus (2/2)	Segniliparus rotundus	WP_013138384	---A--L---A-S----A	--	-TA--I-A----------------
	Segniliparus rugosus	WP_007471275	---A--L---A-S----S	-A	-AA--I-Q----------------
Skermania (1/1)	Skermania piniformis	WP_066469753	--TAT-L---A-V----S	--	-SA--I------------------
Smargdicoccus (1/1)	Smaragdicoccus niigatensis	WP_018162537	--TAT-L---A-V----T	--	-PA----A----------------
Tomitella (1/1)	Tomitella biformata	WP_024796412	--TAT-L---A-V----T	--	-SA----A----------------
Tsukamurella (5/5)	Tsukamurella paurometabola	WP_013126693	--TAT-L---A-V----T	--	-DQ----A----------------
	Tsukamurella pseudospumae	WP_068569466	--TAT-L---A-V----T	--	-DQ----A----------------
	Tsukamurella pulmonis	WP_068534701	--TAT-L---A-V----T	--	-DQ----A----------------
	Tsukamurella sp. 1534	WP_019203041	--TAT-L---A-V----T	--	-DQ----A----------------
	Tsukamurella tyrosinosolvens	WP_068525454	--TAT-L---A-V----T	--	-DQ----A----------------
Williamsia (7/7)	Williamsia faeni	WP_110259832	--TAT-L---A-V----S	--	-PA---------------------
	Williamsia herbipolensis	WP_045822711	--TAT-L---A-V----S	V-	-PGI-I------------------
	Williamsia limnetica	WP_110467681	--TAT-L---A-V----S	--	-PA---------------------
	Williamsia sp. 1135	WP_084896919	--TAT-L---A-V----S	--	-PA---------------------
	Williamsia sp. 1138	WP_084836887	--TAT-L---A-V----S	--	-PA---------------------
	Williamsia sp. Leaf354	WP_055788565	--TAT-L---A-V----S	V-	-PGI-I------------------
	Williamsia sterculiae	WP_076477861	--TAT-L---A-V----S	--	-PA--I------------------
Lawsonella (1/1)	Lawsonella clevelandensis	PZP89957	--TFT-L---A-V----T	V	PGS-V-HE----------------
	Kroppenstedtia sanguinis	WP_124248863	--TAT-L---A------S	--	-SA--IG-----------------

Other Bacteria (1/>1000)

Actinoalloteichus cyanogriseus	WP_081893494	--SAT-L-L-A-S-I--A	DAT---------------------
Actinoalloteichus hoggarensis	WP_093941421	--TAT-L-L-A-S-I--A	-AT----G----------------
Actinoalloteichus hymeniacidon	WP_069848855	--TAT-L-L-A-S-I--S	EAN----A---V------------
Actinoalloteichus spitiensis	WP_026066074	--SAT-L-L-A-S-I--A	DAT---------------------
Actinocatenispora sera	WP_030447513	--TVSGL-L-A-S-I--S	DTA---------------------
Actinocorallia herbida	WP_123663838	--IAK-I-L-A---L-I-	NVTF-IN----V------------
Actinocorallia populi	WP_106402698	--IA--L-L-A-S-L-IE	KATF--N----V------------
Actinokineospora auranticolor	WP_104480081	--TAT-L-L-A-S-I--S	DAS----A----------------
Actinokineospora bangkokensis	WP_075974177	--TAT-L-L-A-S-V--A	DAT---------------------
Actinokineospora enzanensis	WP_018680688	--TVT-L-L-A-S-I--S	-AT---------------------
Actinokineospora inagensis	WP_026425089	--TAT-L-L-A-S-I--A	DAT----A----------------
Actinomadura amylolytica	WP_119730672	--NAT-I-L-A-S-L-IE	KATF--N----V------------
Actinomadura chibensis	WP_067886948	--IAK-I-L-A-S-L-IE	AATF--N----V------------
Actinomadura echinospora	WP_103939270	--NAT-V-L-A-S-L-IE	KATF--N----V------------
Actinomadura flavalba	WP_018656528	--IA--I-L-A-S-L-IE	-ASF--N----V------------
Actinomadura hibisca	WP_067488202	--IA--I-L-A-S-L-IE	-AKF--N----V-F----------
Actinomadura kijaniata	WP_067812066	--IA--I-L-A-S-L-IE	-ATF--N----V-F----------
Actinomadura latina	WP_067638962	--IAK-I-L-A-S-L-IE	AASF--N----V-F----------
Actinomadura oligospora	WP_026410934	--IA--I-L-A---L-IE	-ATF--N----V------------
Actinomadura parvosata subsp.	SPL87570	--IA--I-L-A---L-IE	KASF--N---K-------------
Actinomadura sp. 5-2	WP_103566749	--IA--I-L-A-S-L--E	-VTF--N----V------------
Actinomadura sp. LHW63021	WP_111869074	--IAT-V-L-A-S-L-IE	-AAF--N----V------------
Actinomadura sp. NEAU-G17	WP_117360228	--IA--I-L-A---L-IE	-ATF--N----V------------
Actinomadura sp. NEAU-Ht49	WP_122197940	--IA--I-L-A---L-IE	-ATF--N----V------------
Actinomadura umbrina	WP_116026179	--TAT-V-L-A-S-L-IE	KAAF--N----V------------
Actinomycetospora chiangmaiens	WP_018333595	--TAT-L-L-A-S-V--G	-VT---------AF----------
Actinomycetospora cinnamomea	WP_116707460	--TAS-L-L-A-S-V--S	-AT---------------------
Actinophytocola xanthii	WP_075123505	--TAT-L-L-A-T-I--S	DTT---------------------
Actinophytocola xinjiangensis	WP_075130735	--TAT-L-L-A-T-I--S	DTT---------------------
Actinoplanes atraurantiacus	WP_097322432	--TATGL-L-A---I--S	PVT---------------------
Actinoplanes awajinensis	WP_067704113	--TATGL-L-A-S-I-IS	PTT---------------------
Actinoplanes derwentensis	WP_092546767	--TATGL-L-A---I-IS	PTT---------------------
Actinoplanes friuliensis	WP_023360328	--TATGL-L-A-S-I-IS	PTT---------------------
Actinoplanes globisporus	WP_020510160	--TATGL-L-A---I--S	PTT---------------------
Actinoplanes italicus	WP_106331109	--TATGL-L-A---I-IS	PTT---------------------
Actinoplanes lutulentus	WP_111650154	--TATGL-L-A---I-IS	PTT---------------------
Actinoplanes missouriensis	WP_014441758	--TATGL-L-A---I-IS	PTT---------------------
Actinoplanes philippinensis	WP_093610374	--TATGL-L-A---I-IS	PTT---------------------
Actinoplanes rectilineatus	WP_045741233	--TATGL-L-A-S-I-IS	PTT---------------------
Actinoplanes regularis	WP_089292182	--TATGL-L-A---I-IS	PTT---------------------
Actinoplanes sp. N902-109	WP_015620264	--TATGL-L-A-S-I-IS	PTT---------------------
Actinoplanes sp. TFC3	WP_067498498	--TATGL-L-A-S-I-IS	PTT---------------------
Actinoplanes subtropicus	WP_030439335	--TATGL-L-A---I--S	PTT---------------------
Actinoplanes teichomyceticus	WP_122977196	--TATGL-L-A---I--S	PTT---------------------
Actinoplanes utahensis	WP_043521543	--TATGL-L-A---I-IS	PTT---------------------
Actinoplanes xinjiangensis	WP_109602477	--TATGL-L-A---I-IS	PTT---------------------
Actinopolymorpha alba	WP_020573218	--TASNL-L-A---L--S	-VSF--S---KV------------
Actinopolymorpha singaporensis	WP_092655999	--TVSSL-L-A---L--S	DVSF--A----V------------
Actinopolyspora alba	WP_092927889	--SASGL-L-A-S-I--A	DTS--------V------------
Actinopolyspora erythraea	WP_043574780	--SA-GL-L-A-S-I--E	DTS--------V------------
Actinopolyspora halophila	WP_017975888	--SA-GL-L-A-S-I--S	DAA--------V-------T----
Actinopolyspora mortivallis	WP_019853766	--SA-GL-L-A-S-I--S	DAT--------V------------
Actinopolyspora mzabensis	WP_092627708	--SASGL-L-A-S-I--A	DTS--------V------------
Actinopolyspora righensis	WP_092973450	--SASGL-L-A-S-I--A	DTS--------V------------
Actinopolyspora saharensis	WP_092520584	--SA-GL-L-A-S-I--S	DAA--------V-------T----
Actinopolyspora xinjiangensis	WP_092603853	--SA-GL-L-A-S-I--E	DTS--------V------------
Actinospica robiniae	WP_034269516	--TASHL-------V--Q	DASF--G-----------------
Actinosynnema pretiosum	WP_096496033	--TAT-L-L-A-S-I--S	DAT---------------------
Actinosynnema sp. ALI-1.44	WP_076985617	--TAI-L-L-A-S-I--S	ETT---------------------
Actinotalea fermentans ATCC 43	KGM17106	--TAQ-V-L---V-Q--V	PTSF-IA----V------------
Alloactinosynnema album	WP_091382463	--TAT-L-L-A-S-I--S	DAT--I------------------
Alloactinosynnema iranicum	WP_091447272	--TAT-L-L-A-S-I--S	DAT----A----------------
Alloactinosynnema sp. L-07	WP_054046465	--TAT-L-L-A-S-I--S	DAT--I-A----------------
Allokutzneria albata	WP_030429268	--TVT-L-L-A-S-I--S	DTT---------------------
Allokutzneria sp. NRRL B-24872	WP_086830011	--TVT-L-L-A-S-I--S	DTT---------------------
Allonocardiopsis opalescens	WP_106245860	--TAT-V-L-A-S-L-IE	ATSF--AA---V------------
Alloscardovia macacae	WP_086105790	-SAQGL-I-I---Q--A	PTDFH-S-----------------
Amycolatopsis alba	WP_020637139	--TASGLQL-A-S-I---	DTNV-I-Q----------------
Amycolatopsis albispora	WP_113691252	--TASGL-L-A-S-I--A	DTS--I-Q----------------
Amycolatopsis antarctica	WP_094863439	--TA-GI-L-A-S-I--S	DTS--I-S----------------
Amycolatopsis australiensis	WP_072477999	--TATGL-L-A-S-I---	-AT--I------------------
Amycolatopsis azurea	WP_005152174	--TASGLQL-A-S-I---	DTNV-I-Q----------------
Amycolatopsis balhimycina	WP_020645324	--TATGL-L-A-S-I---	-AT--I------------------
Amycolatopsis benzoatilytica	WP_020658193	--TATGL-L-A-S-I---	-AT--I-S----------------

Other Bacteria
(1/>1000)

Amycolatopsis circi	WP_116206700	--TATGL-L-A-S-I--N	-AT--I-S----------------
Amycolatopsis coloradensis	WP_076165705	--TASGLQL-A-S-I---	ETNV-I-Q----------------
Amycolatopsis decaplanina	WP_007029726	--TASGLQL-A-S-I---	DTNV-I-Q----------------
Amycolatopsis halophila	WP_034270900	--SATGL-L-A-S-I--S	ETA--I------------------
Amycolatopsis jejuensis	WP_033292727	--TATGL-L-A-S-I---	-AT--I-S----------------
Amycolatopsis kentuckyensis	WP_086847878	--TATGL-L-A-S-I--N	-VT--I------------------
Amycolatopsis keratiniphila	WP_016333192	--TASGLQL-A-S-I---	DTNV-I-Q----------------
Amycolatopsis lexingtonensis	WP_086858411	--TATGL-L-A-S-I--N	-VTV-I------------------
Amycolatopsis lurida	WP_034311834	--TASGLQL-A-S-I---	DTNV-I-Q----------------
Amycolatopsis marina	WP_091672909	--TASNL-L-A-S-I--A	DTT--I-Q----------------
Amycolatopsis mediterranei	WP_013224728	--TATGL-L-A-S-I---	-VT--I------------------
Amycolatopsis methanolica	WP_017982989	--TATGL-L-A-S-I--S	DTT--I------------------
Amycolatopsis nigrescens	WP_020672754	--TATGL-L-A-S-I--S	DTT--I-A----------------
Amycolatopsis niigatensis	WP_101438614	--TATGL-L-A-S-I--N	-AT--I-S----------------
Amycolatopsis orientalis	WP_037321059	--TASGLQL-A-S-I---	NTNV-I-Q----------------
Amycolatopsis palatopharyngis	WP_116052004	--TGSNL-L-A-S-I--A	DTT--I-Q----------------
Amycolatopsis pretoriensis	WP_086672474	--TATGL-L-A-S-I--N	-VTV-I-S----------------
Amycolatopsis regifaucium	WP_061980726	--TASGLQL-A-S-I---	DTNV-I-Q----------------
Amycolatopsis rifamycinica	WP_043788220	--TATGL-L-A-S-I--N	-VTV-I-S----------------
Amycolatopsis rubida	WP_093574314	--TATGL-L-A-S-I--N	-AT--I-S----------------
Amycolatopsis saalfeldensis	WP_091618936	--TATGL-L-A-S-I--N	-ATV-I------------------
Amycolatopsis sacchari	WP_091507407	--TATGL-L-A-S-I--A	DTT--I------------------
Amycolatopsis sp. ATCC 39116	WP_020421251	--TATGL-L-A-S-I--S	DTT--I------------------
Amycolatopsis sp. BJA-103	WP_101606817	--TASGLQL-A-S-I---	ETNV-I-Q----------------
Amycolatopsis sp. CA-126428	WP_103336174	--TATGL-L-A-S-I--N	-VT--I------------------
Amycolatopsis sp. CA-128772	WP_103354697	--TATGL-L-A-S-I--N	-VT--I------------------
Amycolatopsis sp. H5	WP_093952050	--TATGL-L-A-S-I---	-AT--I------------------
Amycolatopsis sp. M39	WP_067579130	--TATGL-L-A-S-I--N	-AT--I-S----------------
Amycolatopsis sp. MJM2582	WP_037344071	--TASGLQL-A-S-I---	DTNV-I-Q----------------
Amycolatopsis sp. YIM PH 21725	WP_120025615	--TATGL-L-A-S-I--N	-AT--I-S----------------
Amycolatopsis sulphurea	WP_098509434	--TATGL-L-A-S-I--N	-AT--I-S----------------
Amycolatopsis taiwanensis	WP_027940650	--TATGL-M-A-S-I--S	DTTV-I------------------
Amycolatopsis thailandensis	WP_093934133	--TASGLQL-A-S-I---	DTNV-I-Q----------------
Amycolatopsis tolypomycina	WP_091308648	--TATGL-L-A-S-I--N	-VT--I------------------
Amycolatopsis vancoresmycina	WP_003071241	--TATGL-L-A-S-I---	-AT--I------------------
Amycolatopsis xylanica	WP_091292637	--TATGL-L-A-S-I--S	DTT--I-Q----------------
Angustibacter sp. Root456	WP_056675071	--TVT-L-------V-V-	-ATF--AD---V------------
Antricoccus suffuscus	WP_106347865	--TAEKL-L-A---I-IS	DATM---Q----------------
Arsenicicoccus bolidensis	WP_029210062	--AAT-I-L-A---I-IE	SASF--GA----------------
Arthrobacter alpinus	WP_062006569	--TVQ-L-L-A---L-M-	EVSF-IDK--K-------------
Arthrobacter castelli	WP_026818500	--SVSNL-L-A---L-M-	-VSF-IDK--K-------------
Arthrobacter crystallopoietes	WP_005266749	--TVS-L-L-A---L-ME	AVSF--DK--K-------------
Arthrobacter enclensis	WP_058267287	--TVQ-L-L-A---L-M-	QVSF-IDK--K-------------
Arthrobacter humicola	WP_116765723	--TVQ-L-L-A---L-M-	QVSF-IDK--K-------------
Arthrobacter nitrophenolicus	WP_009358272	--TVQ-L-L-A---L-M-	QVSF-IDK--K-------------
Arthrobacter oryzae	WP_120950415	--TVQ-L-L-A---L-M-	QVSF-IDK--K-------------
Arthrobacter sp. 131MFCol6.1	WP_018772965	--TVQ-L-L-A---L-M-	QVSF-IDK--K-------------
Arthrobacter sp. 162MFSha1.1	WP_018771170	--TVQ-L-L-A---L-M-	QVSF-IDK--K-------------
Arthrobacter sp. 31Y	WP_024816682	--TVQ-L-L-A---L-M-	QVNF--DK--K-------------
Arthrobacter sp. 49Tsu3.1M3	WP_079549596	--TVQ-L-L-A---L-M-	QVSF-IDK--K-------------
Arthrobacter sp. 4R501	WP_104061449	--TVQ-L-L-A---L-M-	QVSF-IDK--K-------------
Arthrobacter sp. 7749	ASN39506	--SVA-L-L-A---L-ME	-VSF-IDK--K-------------
Arthrobacter sp. 9MFCol3.1	WP_026538746	--TVQ-L-L-A---L-M-	QVSF-IDK--K-------------
Arthrobacter sp. AFG7.2	WP_102975521	--TVQ-L-L-A---L-M-	QVSF-IDK--K-------------
Arthrobacter sp. AQ5-05	WP_113762147	--SVA-L-L-A---L-ME	-VSF-IDK--K-------------
Arthrobacter sp. AQ5-06	WP_111908065	--TVQ-L-L-A---L-M-	QVSF-IDK--K-------------
Arthrobacter sp. DCT-5	WP_113717881	--TVQ-L-L-A---L-M-	EVSF-IDK--K-------------
Arthrobacter sp. EPSL27	WP_066438073	--TVQ-L-L-A---L-M-	QVSF-IDK--K-------------
Arthrobacter sp. ERGS1:01	WP_054011129	--TVQ-L-L-A---L-M-	EVSF-IDK--K-------------
Arthrobacter sp. EpRS71	WP_062068715	--TVQ-L-L-A---L-M-	QVNF--DK--K-------------
Arthrobacter sp. FB24	WP_011691960	--TVQ-L-L-A---L-M-	QVSF-IDK--K-------------
Arthrobacter sp. GMC3	WP_104092006	--TVQ-L-L-A---L-M-	EVSF-IDK--K-------------
Arthrobacter sp. H14	WP_026533972	--TVSNL-L-A---L-M-	-VSF-IDK--K-------------
Arthrobacter sp. H20	WP_026552302	--TVSNL-L-A---L-M-	AVSF--DQ--K-------------
Arthrobacter sp. HMWF013	WP_108599562	--TVQ-L-L-A---L-M-	QVSF-IDK--K-------------
Arthrobacter sp. I3	WP_028275532	--TVQ-L-L-A---L-M-	QVSF-IDK--K-------------
Arthrobacter sp. KBS0703	WP_078029388	--TVQ-L-L-A---L-M-	QVSF-IDK--K-------------
Arthrobacter sp. Leaf137	WP_056079041	--TVQ-L-L-A---L-M-	QVSF-IDK--K-------------
Arthrobacter sp. Leaf337	WP_055805845	--TVQ-L-L-A---L-M-	QVSF-IDK--K-------------
Arthrobacter sp. Leaf69	WP_056429341	--TVQ-L-L-A---L-M-	QVSF-IDK--K-------------
Arthrobacter sp. MYb227	WP_105551382	--SVA-L-L-A---L-ME	-VSF-IDK--K-------------
Arthrobacter sp. OV608	WP_091418804	--TVQ-L-L-A---L-M-	QVSF-IDK--K-------------
Arthrobacter sp. PGP41	WP_104997928	--TVQ-L-L-A---L-M-	QVSF-IDK--K-------------
Arthrobacter sp. PM3	AXJ11084	--TVQ-L-L-A---L-M-	QVSF-IDK--K-------------

Other Bacteria (1/>1000)

Arthrobacter sp. RC1.1 241	WP_110543057	--TVQ-L-L-A---L-M-	QVSF-IDK--K------------
Arthrobacter sp. SPG23	WP_043481529	--TVQ-L-L-A---L-M-	QVSF-IDK--K------------
Arthrobacter sp. Soil736	WP_056627101	--TVQ-L-L-A---L-M-	QVSF-IDK--K------------
Arthrobacter sp. Soil761	WP_056390270	--TVQ-L-L-A---L-M-	QVSF-IDK--K------------
Arthrobacter sp. Soil764	WP_056333766	--TVQ-L-L-A---L-M-	QVSF-IDK--K------------
Arthrobacter sp. TB 26	WP_024365669	--TVQ-L-L-A---L-M-	QVSF-IDK--K------------
Arthrobacter sp. U41	WP_069951680	--TVQ-L-L-A---L-M-	QVSF-IDK--K------------
Arthrobacter sp. UCD-GKA	WP_071214947	--SVA-L-L-A---L-ME	-VSF-IDK--K------------
Arthrobacter sp. UNC362MFTsu5.	WP_028269275	--TVQ-L-L-A---L-M-	QVSF-IDK--K------------
Arthrobacter sp. Y81	WP_104174208	--TVQ-L-L-A---L-M-	QVSF-IDK--K------------
Arthrobacter sp. YN	WP_089595084	--TVQ-L-L-A---L-M-	QVNF--DK--K------------
Arthrobacter sp. ZGTC131	WP_104138616	--TVQ-L-L-A---L-M-	QVSF--DK--K------------
Arthrobacter sp. ZGTC412	WP_104043924	--TVQ-L-L-A---L-M-	QVSF-IDK--K------------
Arthrobacter sp. ok362	WP_091561586	--TVQ-L-L-A---L-M-	QVSF-IDK--K------------
Arthrobacter sp. ok909	WP_091254091	--TVQ-L-L-A---L-M-	QVSF-IDK--K------------
Arthrobacter sp. ov118	WP_090956059	--TVQ-L-L-A---L-M-	QVSF-IDK--K------------
Arthrobacter sp. ov407	WP_090577799	--TVQ-L-L-A---L-M-	QVSF-IDK--K------------
Asanoa ferruginea	WP_116074498	--TATGL-L-A-S-I--S	DTT--------------------
Asanoa hainanensis	WP_089253830	--TVTGL-L-A---I--S	DTT--------------------
Asanoa ishikariensis	WP_090790893	--TVTGL-L-A---I--S	DTT--------------------
Bacillus sp. SRB_336	WP_113729387	--TVQ-L-L-A---L-M-	EVSF-IDK--K------------
Blastococcus aggregatus	WP_097195391	--T-TGL-L-A---I-IS	EAN--------------------
Blastococcus atacamensis	WP_104524390	--T-TGL-L-A---I-IS	EAT--------------------
Blastococcus endophyticus	WP_091939307	--T-TGL-L-A---I-IS	-AD--------------------
Blastococcus litoris	WP_116450445	--T-TGL-L-A-S-I-IS	-AD--------------------
Blastococcus saxobsidens	WP_014376959	--T-TGL-L-A---I-IS	EAN-------------------
Blastococcus sp. DSM 44205	SDE62958	--T-TGL-L-A---I-IS	EAN--------------------
Blastococcus sp. DSM 44268	WP_091763700	--T-TGL-L-A---I-IS	EAN--------------------
Blastococcus sp. DSM 44270	WP_097183751	--T-TGL-L-A---I-IS	EAN--------------------
Blastococcus sp. DSM 44272	WP_089334909	--T-TGL-L-A---I-IS	DAD--------------------
Blastococcus sp. DSM 46786	WP_091930510	--T-TGL-L-A---I-IS	EAN--------------------
Blastococcus sp. DSM 46838	WP_092203756	--T-TGL-L-A---I-IS	DAD--------------------
Blastococcus sp. Marseille-P57	WP_106847929	--NAEKI-L-A---V-IS	DATM-------------------
Blastococcus sp. TBT05-19	WP_113812992	--T-TGL-L-A---I-IS	EAT--------------------
Blastococcus sp. TF02-8	WP_113796747	--T-TGL-L-A---I-IS	EAN--------------------
Blastococcus sp. TF02-9	WP_113835651	--T-TGL-L-A---I-IS	-AD--------------------
Blastococcus sp. TF02A-26	WP_113837774	--T-TGL-L-A-S-I-IS	-AD--------------------
Blastococcus sp. TF02A-30	WP_113829375	--T-TGL-L-A---I-IS	EAT--------------------
Blastococcus sp. URHD0036	WP_029431840	--T-TGL-L-A---I-IS	-AD--------------------
Brachybacterium endophyticum	WP_109276168	--TVS-L-I-L---Q-MS	-VSFQ-S----------------
Branchiibius hedensis	WP_109688658	---AT-I-L-A-S-L--S	DASF--AA---------------
Candidatus Blastococcus massil	WP_040339674	--T-TGL-L-A---I-IS	EAD--------------------
Candidatus Frankia californien	SBW22419	--IVS-L-L-A-----VE	AVSF-I-----------------
Candidatus Streptomyces philan	WP_114020831	--TATGL-L-A---I-IE	SATF-IAK---------------
Catellatospora citrea	WP_120315962	--TATGL-L-A---I--G	DTT--------------------
Catelliglobosispora koreensis	WP_020521115	--SA-GL-L-A---I--E	-AN--------------------
Catenulispora acidiphila	WP_012786482	--TAT-V-L-A-S-I-IE	KASF--G----------------
Catenuloplanes japonicus	WP_033340390	--TATGL-L-A---I--S	EAN--I-----------------
Cellulomonas carbonis	WP_043602940	--TAH-V-L---V-V--T	SASF-IG----------------
Cellulomonas sp. NEAU-YY56	WP_122148061	--TAQGV-L-----V--S	EATF--A----------------
Couchioplanes caeruleus	WP_071803531	--TATGL-L-A---I--S	PAT--------------------
Crossiella equi	WP_086780856	--TATGL-L-A-S-I--S	EAN--------------------
Cryptosporangium arvum	WP_035868298	--TVTGL-L-A---I--S	DTT--------------------
Cryptosporangium aurantiacum	WP_073251328	--TVTGL-L-A---I--S	DTT--------------------
Dactylosporangium aurantiacum	WP_033359766	--T-TGL-L-A---I--S	DIN--I-----------------
Demequina salsinemoris	WP_062202502	---AQ-L-M-I---V--H	PASFQ-G----V-----------
Demequina sp. NBRC 110054	WP_084126342	---AQ-L-M-I---V--H	PASFQ-G----V-----------
Demetria terragena	WP_018156065	--TAT-I-L-A-S-L--S	-ATF--A----V-----------
Dermabacter hominis	WP_101686366	--TVH-L-I-----M-MS	-VDFQ-AK---V-----------
Dermabacter jinjuensis	ATH96535	--TVH-L-I-----M-MS	-VDFQ-AK---V-----------
Dermacoccus nishinomiyaensis	WP_038568265	--TAEGI-L-A-S-I--E	NATF--A----V-----------
Dermatophilus congolensis	WP_115030497	-LTAS-I-L-A--QL--R	-ATF--AA---------------
Frankia alni	WP_011605662	--IVSEL-L-A-----IE	PVSF---Q---------------
Frankia asymbiotica	WP_076814780	--IAT-L-L-A---I-VE	PVSF-------------------
Frankia casuarinae	WP_085003061	--IVSEL-L-A-----IE	PVSF----N--------------
Frankia discariae	WP_018502622	--IVSEL-L-A-----IE	PVSF-------------------
Frankia elaeagni	WP_018635500	--IVSEL-L-A-----IE	PVSF-------------------
Frankia inefficax	WP_013425948	--IAA-L-L-A-----VE	PVSF-I-----------------
Frankia sp. AvcI1	WP_055751302	--IVSEL-L-A-----IE	PVSF---Q---------------
Frankia sp. BMG5.30	WP_076842744	--TAS-L-L-A-----IE	AVSF-I-----------------
Frankia sp. BMG5.36	WP_071046906	--IAT-L-L-A---I-VE	PVSF----A--------------
Frankia sp. Cc1.17	WP_071081749	--IVSEL-L-A-----IE	PVSF-------------------
Frankia sp. CcI156	WP_076804415	--IVSEL-L-A-----IE	PVSF----N--------------
Frankia sp. DC12	WP_045875352	--IATGL-L-A-----VE	PVSF-------------------

Other Bacteria (1/>1000)

Frankia sp. EAN1pec	WP_020459805	--IVSEL-L-A-----IE	PVSF--------------------
Frankia sp. EI5c	WP_066067464	--IVSEL-L-A-----IE	PVSF--------------------
Frankia sp. Iso899	WP_022911867	--TAT-L-L-A---I--N	PTT---------------------
Frankia sp. Iso899	WP_022911867	--TAT-L-L-A---I--N	PTT---------------------
Frankia sp. KB5	WP_085014390	--IVSEL-L-A-----IE	PVSF----N---------------
Frankia sp. QA3	WP_009399482	--IVSEL-L-A-----IE	PVSF---Q----------------
Friedmanniella luteola	WP_091410960	-L-AK-L---A---L--E	-VSFQ-A---K-------------
Geodermatophilaceae bacterium	WP_026844234	--T-TGL-L-A---I-IS	DAD---------------------
Geodermatophilus africanus	WP_091150988	--T-TGL-L-A---V-IS	DAN---------------------
Geodermatophilus amargosae	WP_093578675	--T-TGL-L-A---I--S	DVT----A----------------
Geodermatophilus chilensis	WP_100497586	--T-TGL-L-A---V-IS	DAT---------------------
Geodermatophilus nigrescens	WP_073420259	--T-TGL-L-A---I--S	DIS----A----------------
Geodermatophilus normandii	WP_110006095	--T-TGL-L-A---I--G	DVS---------------------
Geodermatophilus obscurus	WP_012948196	--T-TGL-L-A---V-IG	NAN---------------------
Geodermatophilus poikilotrophu	WP_091439504	--T-TGL-L-A---V-IG	NAN---------------------
Geodermatophilus pulveris	WP_089304832	--T-TGL-L-A---V-IS	DAS---------------------
Geodermatophilus ruber	WP_091324419	--T-TGL-L-A---I-IS	EAT---------------------
Geodermatophilus sabuli	WP_097205976	--T-TGL-L-A---I-IS	EAT---------------------
Geodermatophilus saharensis	WP_089405645	--T-TGL-L-A---I--S	DIS----A----------------
Geodermatophilus siccatus	WP_091217187	--T-TGL-L-A---V-IS	DAD---------------------
Geodermatophilus sp. DSM 44208	WP_091111096	--T-TGL-L-A---I--G	DVT---------------------
Geodermatophilus sp. DSM 44511	WP_110907336	--T-TGL-L-A---I--S	DVS---------------------
Geodermatophilus sp. DSM 45219	WP_091376093	--T-TGL-L-A---V-IS	DAT---------------------
Geodermatophilus sp. LHW52908	WP_117379637	--T-TGL-L-A---V-IS	EAD---------------------
Geodermatophilus sp. Leaf369	WP_055766763	--T-TGL-L-A-S-I-IS	EAD---------------------
Geodermatophilus sp. TF02-6	WP_113779828	--T-TGL-L-A---V-IS	DAT---------------------
Geodermatophilus telluris	WP_091365864	--T-TGL-L-A---I--G	DVT---------------------
Geodermatophilus tzadiensis	WP_106276654	--T-TGL-L-A---I--G	DVT---------------------
Glycomyces arizonensis	WP_026922992	--TATGL-L-A-S-I--S	-AD---------------------
Glycomyces artemisiae	WP_106364492	--TATGL-L-A-S-I-VS	-AD---------------------
Glycomyces dulcitolivorans	WP_112139402	--TATGL-L-A-S-I--S	-AD---------------------
Glycomyces harbinensis	WP_091034006	--TAAGL-L-A-S-I--S	-AD--I------------------
Glycomyces sambucus	WP_091054286	--TATGL-L-A-S-I--S	-AD---------------------
Glycomyces sp. NRRL B-16210	WP_030161352	--TATGL-L-A-S-I--S	-AD--I------------------
Glycomyces sp. YIM 121974	RRS00657	--TATGL-L-A-S-I--S	-AD---------------------
Glycomyces tenuis	WP_026930604	--TATGL-L-A-S-I--S	-AD---G-----------------
Glycomyces xiaoerkulensis	WP_100444983	--TASGL-L-A---I--T	-AD----------------S---S
Haloactinopolyspora alba	WP_106537380	--TATQV-L-A---I---	DASF-IN---KV------------
Haloechinothrix alba	WP_089302696	--TATGL-L-A-S-I--T	DTA--------------------S
Haloglycomyces albus	WP_025274489	--QAT-LQL-A-S-I--E	EVS----A---V-----------S
Halopolyspora algeriensis	WP_114454219	--SASGL-L-A-S-I--S	DAS--------V------------
Hamadaea tsunoensis	WP_027343423	--TATGL-L-A-S-I--S	DAL---------------------
Helcobacillus massiliensis	WP_055088056	--TVH-L-I-----M-MS	-VNFQ-AK---V------------
Herbidospora cretacea	WP_061292526	--IAT-I-L---S-L-IE	-ASF--N---K-------------
Herbidospora daliensis	WP_062437687	--IAT-I-L---S-L-IE	-ASF--N---K-------------
Herbidospora mongoliensis	WP_066368164	--IAT-I-L---S-L-IE	-ASF--N---K-------------
Herbidospora sakaeratensis	WP_062340351	--IAT-I-L---S-L-IE	-ASF--N---K-------------
Herbidospora yilanensis	WP_062348566	--IAT-I-L---S-L-IE	-ASF--N---K-------------
Isoptericola variabilis	WP_013838535	--TAH-V-L-----V--H	-ATF-ISA----------------
Janibacter hoylei PVAS-1	EKA61027	--TASGI-L-A-S-I--E	-ASF--AA---V------------
Janibacter indicus	WP_072624663	--TASGI-L-A-S-I--E	-ASF--A----V------------
Janibacter limosus	WP_068258287	--TASSI-L-A-S-I---	-ASF-MAA---V------------
Janibacter melonis	WP_068275940	--TATSV-L-A-S-I--E	EASF--AA---V------------
Janibacter sp. HTCC2649	WP_009774973	-LTAAAI-L-A-S-L---	NVSF---A----------------
Janibacter sp. Marseille-P4121	WP_106820021	---ASGI-L-A-S-I---	DASF--A----V------------
Janibacter sp. Soil728	WP_055995585	--TASSI-L-A-S-I---	-ASF-MAA---V------------
Jatrophihabitans endophyticus	WP_073390955	--TAH-L-L-A---I--E	PTT----A----------------
Jatrophihabitans sp. GAS493	WP_096303798	--TASGL-L-A---I--E	PTN--I------------------
Jiangella alba	WP_069115001	--TAHQL---A--QI--E	DASF-IG---KV------------
Jiangella alkaliphila	WP_046769435	--TAHHL---A--QV---	DAS--IG---KV------------
Jiangella gansuensis	WP_026875888	--TAHQL---A--QI--S	DASF-IA---K-------------
Jiangella muralis	WP_053202383	--TAHQL---A--QI--E	DASF-IG---KV------------
Jiangella sp. DSM 45060	WP_092619737	--TAHQL---A--QI--E	DASF-IG---KV------------
Jiangella sp. GTF31	WP_111257321	--TAHHL---A--QV--E	DASF-IG---K-------------
Jiangella sp. KE2-3	WP_116950692	--TAHQL---A--QI--E	DASF-IG---KV------------
Jiangella sp. NEAU-YY265	WP_119659662	--TAHQL---A--QV--E	DASF-IG---KV------------
Jishengella endophytica	WP_111243025	--TASGL-L-A---I--S	DTT---------------------
Jishengella sp. AZ1-13	WP_119578059	--TASGL-L-A---I--S	DTT---------------------
Jishengella sp. NA12	WP_111215361	--TASGL-L-A---I--S	DTT---------------------
Jonesia denitrificans	WP_015771444	--TATGV-L-I------Q	PTSF--G-----------------
Kibdelosporangium aridum	WP_084424837	--TAI-L-L-A-S-I--S	ETT---------------------
Kibdelosporangium phytohabitan	WP_054290918	--TAI-L-L-A-S-I--S	ETT---------------------
Kibdelosporangium sp. MJ126-NF	WP_042192090	--TAI-L-L-A-S-I--S	ETT---------------------
Kineococcus radiotolerans	WP_041292228	---AS-L-L-A---L-ME	-VNF--AA----------------

Other Bacteria (1/>1000)

Kineococcus rhizosphaerae	WP_106206223	---AT-I-L-A---L-ME	-VNF--AA---------------
Kineococcus xinjiangensis	WP_104432525	---AS-I-L-A---L-ME	HVTF--A----V-F---------
Kineosphaera limosa	WP_006591022	--TATGV-L-A-S-L--S	DATF--G----V-----------
Kitasatospora albolonga	WP_084745238	--TATGI-L-A---I-IE	SASF-IAK---------------
Kitasatospora azatica	WP_035841735	--SA-AL-L-A---I-IE	SASF--A----------------
Kitasatospora cheerisanensis	WP_035867218	--SA-AL-L-A---I-IE	SASF-IA----------------
Kitasatospora mediocidica	WP_035794345	--SV-AL-L-A---I-IE	SASF--T----------------
Kitasatospora phosalacinea	WP_033220339	--SA-AL-L-A---I-IE	SASF-IA----------------
Kitasatospora setae	WP_014138755	--SA-AL-L-A---V-IE	SASF-IA----------------
Kitasatospora sp. CB01950	WP_073814659	--SA-AL-L-A---I-IE	SASF-IA----------------
Kitasatospora sp. MBT63	WP_033819317	--SA-AL-L-A---I-IE	SATF-IA----------------
Kitasatospora sp. MMS16-BH015	WP_104818832	--SV-AL-L-A---I-IE	SASF--T----------------
Kitasatospora sp. NRRL B-11411	WP_030461121	--SA-AL-L-A---I-IE	SASF-IA----------------
Kitasatospora sp. OK780	WP_100888060	--SA-AL-L-A---V-IE	SASF--A----------------
Kitasatospora sp. SolWspMP-SS2	WP_111552843	--SA-AL-L-A---I-IE	SASF-IA----------------
Klenkia soli	WP_091247956	--T-TGL-L-A---I-IS	DAN--------------------
Knoellia sinensis	WP_035915566	-LTAASI-L-A-S-L---	-ATF--AA---------------
Kocuria indica	WP_085107472	--TVTNL-L-A---L-M-	EVNF--DK--KV-----------
Kocuria marina	WP_035962025	--TVTNL-L-A---L-M-	EVNF--DK--KV-----------
Kocuria palustris	WP_006213161	--TVSNL-L-A---L-M-	DVDF---K--KV-----------
Kocuria rhizophila	WP_012398303	--TVT-L-L-A---L-M-	EVNF--DK--KV-----------
Kocuria salsicia	WP_055086230	--TVTNL-L-A---L-M-	EVSF--DK--KV-----------
Kocuria sp. 442	WP_121029829	--TVT-L-L-A---L-M-	EVNF--DK--KV-----------
Kocuria sp. BT304	WP_112235081	--TVT-L-L-A---L-M-	EVNF--DK--KV-----------
Kocuria sp. CPCC 104605	WP_119933670	--TVSNL-L-A---L-M-	QVNF---R--K------------
Kocuria sp. ICS0012	WP_064845366	--TVTNL-L-A---L-M-	EVNF--DK--KV-----------
Kocuria sp. M5W7-7	WP_123824784	--TVSNL-L-A---L-M-	QVNF---R--K------------
Kocuria sp. Marseille-P3598	WP_085529484	--TVSNL-L-A---L-M-	EVNF--NK--KV-----------
Kocuria sp. PT10	WP_124109305	--TVT-L-L-A---L-M-	EVNF--DK--KV-----------
Kocuria sp. WRN011	WP_095797887	--TVTNL-L-A---L-M-	EVNF--DR--KV-----------
Kocuria sp. ZOR0020	WP_047690888	--TVSNL-L-A---L-M-	QVNF---R--K------------
Kocuria tytonicola	WP_121845439	--TVTNL-L-A---L-M-	EVNF--DR--KV-----------
Kocuria varians	WP_068467132	--TVTNL-L-A---L-M-	EVNF--DK--KV-----------
Kribbella catacumbae	WP_020384786	--TVTNL-------Q--S	PASF--G---KV-----------
Kribbella flavida	WP_012920920	--TVTNL-------Q--A	PASF--G---KV-----------
Kribbella sp. ALI-6-A	WP_077013940	--TVSNL-------Q--A	PASF--G---KV-----------
Kribbella sp. NEAU-SW521	WP_112236604	--TVSNL-------Q--A	PASF--G---KV-----------
Kutzneria albida	WP_025357224	--TVT-L-L-A-S-I-VS	-AT--------------------
Kutzneria buriramensis	WP_116180713	--TVT-L-L-A-S-I--S	DAT--------------------
Kutzneria sp. 744	EWM10975	L-TVT-L-L-A-S-I--S	DAT--------------------
Kytococcus aerolatus	WP_088817167	--TVDGL-L-A-P-L--E	PTSF---A---------------
Labedella gwakjiensis	WP_106564395	-LSVH-L-I-----V-ME	-VDF--SD--K------------
Lechevalieria aerocolonigenes	WP_045317391	--TAT-M-L-A-S-I-VS	-AN--------------------
Lechevalieria atacamensis	WP_112226161	--TAT-M-L-A-S-I-VS	-AN--------------------
Lechevalieria deserti	WP_109635378	--TAT-M-L-A-S-I-VS	-AN--------------------
Lechevalieria fradiae	WP_090056045	--TAT-M-L-A-S-I-VS	-AN--------------------
Lechevalieria xinjiangensis	WP_089955229	--TAT-M-L-A-S-I-VS	-AN--------------------
Leifsonia xyli	WP_048678263	--TVSNL-L-A---L-M-	EVNF--NK--KV-----------
Lentzea albida	WP_089916810	--TAT-M-L-A-S-I-VS	-AN--------------------
Lentzea albidocapillata	WP_030479125	--TAT-M-L-A-S-I-VS	-AN--------------------
Lentzea flaviverrucosa	WP_090065172	--TAT-M-L-A-S-I-VS	-AN--------------------
Lentzea guizhouensis	WP_065917642	--TAT-M-L-A-S-I-VS	-AN--------------------
Lentzea jiangxiensis	WP_090105500	--TAT-M-L-A-S-I-VS	-AN--------------------
Lentzea kentuckyensis	WP_086668746	--TAT-M-L-A-S-I-VS	-AN--------------------
Lentzea terrae	WP_112269370	--TAT-M-L-A-S-I-VS	-AN--------------------
Lentzea violacea	WP_090010971	--TAT-M-L-A-S-I-VS	-AN--------------------
Lentzea waywayandensis	WP_093604423	--TAT-M-L-A-S-I-VS	-AN--------------------
Longispora albida	WP_026212225	--SVTGL-L-A---I--S	DTT--------------------
Luteipulveratus halotolerans	WP_050669540	--TASGV-L-A---I--E	-ATF--AA---V-----------
Luteipulveratus mongoliensis	WP_052592574	--TATGI-L-A-S-I---	DATF--A----V-----------
Luteococcus japonicus	WP_094766055	-LQVK-L---A---L--S	PISFQ-I----------------
Lysinimicrobium aestuarii	WP_062466223	--IAK-L-I-I---V-VH	PASFQIG----V-----------
Lysinimicrobium flavum	WP_062291987	--IAK-L---I---V--H	PASFQIG----V-----------
Lysinimicrobium gelatinilyticu	WP_062518127	--IAK-L---I---V--H	PASFQIG----V-----------
Lysinimicrobium iriomotense	WP_062388941	--IAK-L---I---V--H	PTSFQIG----V-----------
Lysinimicrobium luteum	WP_062522094	--IAK-L---I---V--H	PASFQIG----V-----------
Lysinimicrobium mangrovi	WP_042215580	--IAK-L-I-I---V-VH	PASFQIG----V-----------
Lysinimicrobium pelophilum	WP_062377554	---AK-L-M-I---V--H	PASFQIG----V-----------
Lysinimicrobium rhizosphaerae	WP_062314789	--IAK-L-I-I---V--H	PASFQIG----V-----------
Lysinimicrobium soli	WP_062464832	--IAK-L---I---V--H	PASFQIG----V-----------
Lysinimicrobium subtropicum	WP_062307158	--IAK-L---I---V--H	PVSFQIG----V-----------
Marinactinospora thermotoleran	WP_078762568	-LIAS-L-I---S-L--E	PTSF--AA---------------
Marmoricola ginsengisoli	WP_123225855	--TASRL---A---L-ME	NVSF-IAT---------------
Marmoricola sp. 4Q3S-7	WP_121806480	--TAQQV---A---L-ME	DVSF--G----V-----------

Other Bacteria (1/>1000)

Marmoricola sp. URHB0036	WP_027860708	--TVSKL---A---L-M-	DVSF-IAA---------------
Microbacterium agarici	WP_098407975	-LNVS-L-I-----V-MQ	NVSF------K------------
Microbispora rosea	WP_030507272	--IAT-I-L-A-S-L-IE	-ASF--N---K------------
Microbispora sp. ATCC PTA-5024	WP_036318542	--IAT-V-L-A-S-L-IE	AAGF--N---K------------
Microbispora sp. GKU 823	WP_079318791	--IAS-I-L-A-S-L-IE	-ASF--N---K------------
Microbispora sp. GMKU363	WP_055482136	--IAT-V-L-A-S-L-IE	-ASF--N---K------------
Microbispora triticiradicis	WP_117410033	--IAT-V-L-A-S-L-IE	-ASF--N---K------------
Micromonospora arborensis	WP_110567858	--TATGL-L-A-S-I--S	DTT--------------------
Micromonospora aurantiaca	WP_123111148	--TATGL-L-A-S-I--S	DTT--------------------
Micromonospora auratinigra	WP_091663265	--TATGL-L-A-S-I--S	DTT--------------------
Micromonospora avicenniae	WP_076467623	--TATGL-L-A---I--S	DTT--------------------
Micromonospora carbonacea	WP_074474321	--TATGL-L-A---I--S	DTT--------------------
Micromonospora chaiyaphumensis	WP_091270081	--NATGL-L-A-S-I--S	DTT--------------------
Micromonospora chersina	WP_091320802	--TATGL-L-A-S-I--S	DTT--------------------
Micromonospora chokoriensis	WP_030491555	--TATGL-L-A-S-I--S	DTT--------------------
Micromonospora citrea	WP_091105261	--TATGL-L-A---I--S	DTT--------------------
Micromonospora coriariae	WP_089019876	--TATGL-L-A-S-I--S	DTT--------------------
Micromonospora costi	WP_120779736	--TATGL-L-A---I--S	DTT--------------------
Micromonospora coxensis	WP_088975110	--TATGL-L-A---I--S	DTT--------------------
Micromonospora cremea	WP_074312754	--TATGL-L-A-S-I--S	DTT--------------------
Micromonospora eburnea	WP_091121741	--TATGL-L-A-S-I--S	DTT--------------------
Micromonospora echinaurantiaca	WP_088992917	--TATGL-L-A---I--S	DTT--------------------
Micromonospora echinofusca	WP_088999385	--TATGL-L-A---I--S	DTT--------------------
Micromonospora echinospora	WP_088983062	--TATGL-L-A---I--S	DTT--------------------
Micromonospora endolithica	WP_120724284	--TASGL-L-A-S-I--S	DTT--------------------
Micromonospora globbae	WP_120327383	--TATGL-L-A---I--S	DTT--------------------
Micromonospora globispora	WP_109965730	--TATGL-L-A-S-I--S	DTT--------------------
Micromonospora haikouensis	WP_091278516	--TATGL-L-A---I--S	DTT--------------------
Micromonospora halophytica	WP_091290947	--TATGL-L-A---I--S	DTT--------------------
Micromonospora humi	WP_091071849	--TATGL-L-A-S-I--S	DTT--------------------
Micromonospora inositola	WP_089013521	--TATGL-L-A-S-I--S	DTT--------------------
Micromonospora inyonensis	WP_091460262	--TATGL-L-A---I--S	DTT--------------------
Micromonospora krabiensis	WP_091597112	--TATGL-L-A---I--S	DTT--------------------
Micromonospora lupini	WP_007462683	--TATGL-L-A-S-I--S	DTT--------------------
Micromonospora matsumotoense	WP_091237315	--SVTGL-L-A-S-I--S	DTT--------------------
Micromonospora mirobrigensis	WP_091615939	--TATGL-L-A-S-I--S	DTT--------------------
Micromonospora narathiwatensis	WP_091195774	--TATGL-L-A-S-I--S	DTT--------------------
Micromonospora nigra	WP_091076178	--TATGL-L-A-S-I--S	DTT--------------------
Micromonospora pallida	WP_091639030	--TATGL-L-A---I--S	DTT--------------------
Micromonospora parva	WP_030337302	--TATGL-L-A-S-I--S	DTT--------------------
Micromonospora pattaloongensis	WP_091557877	--TATGL-L-A---I--S	DTT--------------------
Micromonospora peucetia	WP_091629982	--TATGL-L-A---I--S	DTT--------------------
Micromonospora pisi	WP_121155384	--TVSGL-L-A---I--S	DTT--------------------
Micromonospora purpureochromog	WP_088961267	--TATGL-L-A---I--S	DTT--------------------
Micromonospora rhizosphaerae	WP_091345926	--TATGL-L-A-S-I--S	DTT--------------------
Micromonospora rifamycinica	WP_067304334	--SVTGL-L-A-S-I--S	DTT--------------------
Micromonospora rosaria	WP_067365646	--TATGL-L-A---I--S	DTT--------------------
Micromonospora sediminicola	WP_091573422	--TATGL-L-A-S-I--S	DTT--------------------
Micromonospora siamensis	WP_088972218	--TATGL-L-A-S-I--S	DTT--------------------
Micromonospora sp. 4G51	WP_109805479	--TATGL-L-A-S-I--S	DTT--------------------
Micromonospora sp. 5R2A7	WP_109819596	--TATGL-L-A-S-I--S	DTT--------------------
Micromonospora sp. ATCC 39149	WP_007072024	--TATGL-L-A---I--S	DTT--------------------
Micromonospora sp. BL-1	WP_121685548	--TATGL-L-A-S-I--S	DTT--------------------
Micromonospora sp. BL-4	WP_121676492	--TATGL-L-A-S-I--S	DTT--------------------
Micromonospora sp. CB01531	WP_073837964	--TATGL-L-A-S-I--S	DTT--------------------
Micromonospora sp. CNZ309	WP_101411624	--TATGL-L-A---I--S	DTT--------------------
Micromonospora sp. CV-4	WP_121656687	--TATGL-L-A-S-I--S	DTT--------------------
Micromonospora sp. HK10	WP_046563216	--TATGL-L-A-S-I--S	DTT--------------------
Micromonospora sp. HM5-17	WP_123560870	--TATGL-L-A---I--S	DTT--------------------
Micromonospora sp. LB19	WP_124821547	--TATGL-L-A-S-I--S	DTT--------------------
Micromonospora sp. LB32	WP_124853761	--TATGL-L-A-S-I--S	DTT--------------------
Micromonospora sp. LB39	WP_124774331	--TATGL-L-A-S-I--S	DTT--------------------
Micromonospora sp. Llam0	WP_123602400	--TATGL-L-A-S-I--S	DTT--------------------
Micromonospora sp. M42	WP_043329650	--TATGL-L-A-S-I--S	DTT--------------------
Micromonospora sp. M71_S20	WP_121400702	--TATGL-L-A---I--S	DTT--------------------
Micromonospora sp. MH33	WP_107078996	--TATGL-L-A-S-I--S	DTT--------------------
Micromonospora sp. MW13	WP_117669704	--TATGL-L-A---I--S	DTT--------------------
Micromonospora sp. NBS 11-29	WP_089156119	--TATGL-L-A-S-I--S	DTT--------------------
Micromonospora sp. NRRL B-1680	WP_053657033	--TATGL-L-A-S-I--S	DTT--------------------
Micromonospora sp. PPF5-17	WP_123241421	--TATGL-L-A-S-I--S	DTT--------------------
Micromonospora sp. RP3T	WP_107162512	--TATGL-L-A-S-I--S	DTT--------------------
Micromonospora sp. Rc5	WP_077939177	--TATGL-L-A---I--S	DTT--------------------
Micromonospora sp. S4605	WP_109898085	--TATGL-L-A---I--S	DTT--------------------
Micromonospora sp. WMMA1996	WP_098748201	--TATGL-L-A-S-I--S	DTT--------------------

Other Bacteria (1/>1000)

Micromonospora sp. WMMA2032	WP_099164398	--IATGL-L-A-S-I--S	DTT--------------------
Micromonospora tulbaghiae	WP_120568938	--TATGL-L-A-S-I--S	DTT--------------------
Micromonospora viridifaciens	WP_089006143	--TATGL-L-A-S-I--S	DTT--------------------
Micromonospora wenchangensis	WP_088645037	--SVTGL-L-A-S-I--S	DTT--------------------
Micromonospora yangpuensis	WP_091440688	--TATGL-L-A---I--S	DTT--------------------
Microtetraspora fusca	WP_066936974	--IAT-I-L-A-S-L-IE	-ASF--N---K------------
Microtetraspora glauca	WP_061255602	--IAT-V-L-A-S-L-IE	-ASF--N---K------------
Microtetraspora malaysiensis	WP_067131631	--IAT-I-L-A-S-L-IE	-ASF--N---K------------
Microtetraspora niveoalba	WP_067179299	--IAT-V-L-A-S-L-IE	-ASF--N---K------------
Modestobacter caceresii	WP_036338656	--T-TGL-L-A---I-IS	DAD--------------------
Modestobacter marinus	WP_014741596	--T-TGL-L-A---I-IS	DAD--------------------
Modestobacter sp. DSM 44400	WP_091539685	--T-TGL-L-A---I-IS	DAN--------------------
Modestobacter sp. Leaf380	WP_056300868	--T-TGL-L-A-S-I-IS	EAD--------------------
Modestobacter sp. VKM Ac-2676	OMQ14869	--T-TGL-L-A-S-I-IS	DAD--------------------
Modestobacter versicolor	WP_110553625	--T-TGL-L-A-S-I-IS	EAD--------------------
Mycetocola zhadangensis	WP_121658581	-LTVH-L-I-----V-ME	-VNF--AA--K------------
Mycobacteroides abscessus subs	SIL10859	--TVTNL-L-A---L-M-	EVNF-IDK--K------------
Nakamurella lactea	WP_029135947	--TATGL-L-A-T-I--A	DTT--L-A---V-----------
Nakamurella multipartita	WP_015747556	--TATGL-L-A-T-I--A	DTA---HA---------------
Nakamurella panacisegetis	WP_090479693	--TATGL-L-A-T-I--A	DTTV-L-H---V-----------
Nakamurella silvestris	WP_119386383	--TATGL-L-AAS-I--A	DTSV-I-Q---------------
Nakamurella sp. 12Sc4-1	WP_111765601	--TATGL-L-AAS-I--S	DVN---------AF---------
Nakamurella sp. s14-144	WP_124798962	--TATGL-L-AAS-I--A	DTSV---T----AF---------
Nesterenkonia alba	WP_022872502	--SVSNL-L-A---L-M-	-VSF--DA--K------------
Nesterenkonia massiliensis	WP_044494679	--SVT-L-L-----L-M-	EVNF--DA--K------------
Nesterenkonia sp. M8	WP_119903285	--SVSNL-L-A---L-M-	-VSF-ID---K------------
Nesterenkonia sp. RB2	WP_120005353	--SVSNL-L-A---L-M-	-VSF-IDS--K------------
Nocardioides daejeonensis	WP_110205600	--TAQQL---A---L-ME	NVSF--AA--K------------
Nocardioides jensenii	WP_067430054	--TAQQL---A---L-ME	NVNF--AA--KV-----------
Nocardioides lianchengensis	WP_090856204	--TASQL---A---L-ME	NVSF--AA--KV-----------
Nocardioides luteus	WP_008357082	--TAH-L-------L-ME	NVTF--G---KV-----------
Nocardioides sp. CF167	WP_107777330	--TAHQL-------L-ME	NVSF--G---KV-----------
Nocardioides sp. EGI 63112	WP_121251352	--TAHQL---A---L-ME	NVSF--G---KV-----------
Nocardioides sp. Iso805N	WP_026146032	--TAQNL-------L-ME	NVSF--G---KV-----------
Nocardioides sp. Soil797	KRF17118	--TAQQL---A---L-ME	NVNF--AA--KV-----------
Nocardioides sp. YIM ART13	WP_109508527	--TAQQL---A---L-ME	NVSF--A---KV-----------
Nocardioides sp. YR527	WP_091042352	--TAH-L-------L-ME	NVTF--G---KV-----------
Nocardioides szechwanensis	WP_091023245	--TAHQL---A---L-M-	-VSF--AA--KV-----------
Nocardioides terrae	WP_091123609	--TAHQL-------L-M-	DVSF--G---KV-----------
Nocardiopsis alba	WP_014912012	-LIAT-L-L-----L--E	PTTFQ-AA---------------
Nocardiopsis alkaliphila	WP_017606508	-LIAT-L-L-----L--E	PTTFQ-AA---------------
Nocardiopsis halotolerans	WP_017571525	-LIAT-L-L---P-L--E	PTTF--A----------------
Nocardiopsis listeri	WP_067599737	-LIAT-L-M-----L--E	PTTFQ-AA---------------
Nocardiopsis lucentensis	WP_017601801	-LIAT-L-L---P-V--E	PSTF--AA---------------
Nocardiopsis salina	WP_017613294	-LIAT-L-L---S-L--E	PTSF--AA--K------------
Nocardiopsis sp. CNR-923	WP_075925083	-LIAT-L-L---P-V--E	PSTF--AA---------------
Nocardiopsis sp. CNT312	WP_028649376	-LIAT-L-L-----L--E	PTTF--GA---------------
Nocardiopsis sp. Huas11	WP_121183023	-LIAT-L-L---P-L--E	PTSF--AA---------------
Nocardiopsis sp. JB363	WP_087096501	-LIAT-L-M-----L--E	PTTFQ-AA---------------
Nocardiopsis sp. NRRL B-16309	WP_053619529	-LIAT-L-L---P-L--E	PTSF--AA---------------
Nocardiopsis trehalosi	WP_067974160	-LIAT-L-L---P-L--E	PSSF---A---------------
Nonomuraea candida	WP_043628086	--IA--I-L-A---L-IE	KASF--N---K------------
Nonomuraea coxensis	WP_020547487	--IA--I-L-A---L-IE	KASF--N---K------------
Nonomuraea fuscirosea	WP_106251430	--IA--I-L-A---L-IE	KATF--N---K------------
Nonomuraea gerenzanensis	SBO98147	--IA--I-L-A---L-IE	KATF--N---K------------
Nonomuraea indica	WP_101786874	--IAS-I-L-A---L-IE	SASF--N---K------------
Nonomuraea jiangxiensis	WP_090942789	--IAT-I-L-A---L-IE	KASF--N---K------------
Nonomuraea maritima	WP_090765076	--IAS-I-L-A---L-I-	KASF--N---K------------
Nonomuraea solani	WP_103961814	--IAS-I-L-A---L-IE	KASF--N---K------------
Nonomuraea sp. ATCC 55076	WP_080037136	--IA--I-L-A---L-IE	KASF--N---K------------
Nonomuraea sp. KC333	WP_111176654	--IAS-I-L-A---L-IE	KASF--N---K------------
Nonomuraea sp. NEAU-YG30	WP_113704580	--IAS-I-L-A---L-IE	SASF--N---K------------
Nonomuraea sp. SBT364	WP_049568011	--IAS-I-L-A-S-L-IE	NASF--N---K------------
Nonomuraea wenchangensis	WP_091087368	--IA--I-L-A---L-IE	KASF--N---K------------
Paeniglutamicibacter gangotrie	WP_007271604	--SVA-L-L-A---L-ME	-VSF-IDK--K------------
Phycicoccus jejuensis	WP_030528421	--TAASV-L-A-S-L---	-ATF--A----------------
Phycicoccus sp. Soil748	WP_056885209	--AASGI-L-A-S-L---	-ATF-IA----V-----------
Planobispora rosea	WP_068924103	--IAS-I-L-A---L-IE	-ASF--N---K------------
Planomonospora sphaerica	WP_068899246	--IAS-I-L-A---L-IE	-ASF--N---K------------
Plantactinospora sp. CNZ320	WP_101366993	--TASGL-L-A---I--S	DTT--------------------
Plantactinospora sp. KBS50	WP_095566056	--TATGL-L-A-S-I--S	EAT--------------------
Prauserella marina	WP_091808177	--TA-GL-L-A-S-V--S	DTT--I-----------------
Prauserella muralis	WP_112285031	--TATGL-L-A-S-V--S	-TT--------------------
Prauserella rugosa	WP_030530551	--TATGLQL-A-S-I---	DTT--------------------

Other Bacteria (1/>1000)

Prauserella shujinwangii	WP_106180339	--TATGL-L-A-S-I--S	-TT---------------------
Prauserella sp. Am3	KID30338	--TATGLQL-A-S-I---	DTT---------------------
Prauserella sp. YIM 121212	WP_110344054	--TAT-L-L-A---L--A	-AT---------------------
Propionibacteriaceae bacterium	WP_094363392	-LSVR-L---A---L--S	AVSFQ-T---K-------------
Propionibacterium australiense	WP_119161111	--QA--L-------L--A	PVSF--AK--T--V----------
Propionibacterium freudenreich	SCQ72189	--QAKEL---A---L--N	PVSFQ-I---K--V----------
Propionibacterium sp. oral tax	WP_016670187	-LQAT-L---A---L--E	PTTF--N---K-------------
Propionimicrobium lymphophilum	WP_016455775	-LSA-NL---I---L--E	PTSF-IN---K--F----------
Propionimicrobium sp. BV2F7	WP_024110935	-LSA-NL---I---L--E	PTSF-IN---K--F----------
Propionimicrobium sp. Marseill	WP_076388851	-LQATNL--WI---R--A	PTTF--N---K--F----------
Pseudarthrobacter phenanthreni	WP_013600929	--TVQ-L-L-A---L-M-	QVSF-IDK--K-------------
Pseudarthrobacter siccitoleran	WP_050055311	--TVQ-L-L-A---L-M-	QVSF-IDK--K-------------
Pseudarthrobacter sp. T11b	WP_124092909	--TVQ-L-L-A---L-M-	QVSF-IDK--K-------------
Pseudokineococcus lusitanus	WP_123378242	--TAT-V-L-A---L--G	-ASF--A----V------------
Pseudonocardia acaciae	WP_028923044	--TAT-L-L-A-----VA	-AT---A-----------------
Pseudonocardia ammonioxydans	WP_093344914	--T-T-L-L-A-S-I--S	-AD---------------------
Pseudonocardia asaccharolytica	WP_028929981	--TAT-L-L-A-S-I--S	-AT---------------------
Pseudonocardia autotrophica	WP_037038752	--TAT-L-L-A-S-I--S	-AT---------------------
Pseudonocardia dioxanivorans	WP_013675498	--TAT-M-L-A-S-I--A	-AT---------------------
Pseudonocardia oroxyli	WP_093077810	--TAS-L-L-A-S-I--A	-AN---------------------
Pseudonocardia sp. 73-21	OJY39140	--TAT-L-L-A-S-I--S	-ATM--------------------
Pseudonocardia sp. CNS-004	WP_075954112	--TAS-L-L-A-S-I--S	DAT---------------------
Pseudonocardia sp. HH130629-09	WP_060711930	--T-T-L-L-A-S-I--S	-AT---------------------
Pseudonocardia sp. HH130630-07	WP_068799232	--S-SNI-L-A-S-I--S	-AN---------------------
Pseudonocardia sp. MH-G8	WP_094928929	--TAT-L-L-A-S-I--S	-AT----A----------------
Pseudonocardia sp. N23	WP_098960908	--TAT-L-L-A-S-I--S	-AD---------------------
Pseudonocardia sp. SCN 72-51	ODU10980	--TAT-L-L-A-S-I--S	-AD---------------------
Pseudonocardia sp. SCN 73-27	ODV07084	--TAT-L-L-A-S-I--S	-AD---------------------
Pseudonocardia spinosispora	WP_028932229	--TAT-L-L-A---I-VA	DAN--IA-----------------
Pseudonocardia thermophila	WP_073454973	--TAT-L-L-A-S-V-VS	-AT---------------------
Pseudonocardiaceae bacterium Y	WP_120088525	--SVT-L-L-A-S-I--S	-TT--I------------------
Pseudonocardiales bacterium	PZS02592	--TASAL-L-A-S-I-IG	DAD---------------------
Pseudopropionibacterium propio	RRD51550	-LQVK-V---A---L--S	PATFQIA----V------------
Pseudosporangium ferrugineum	WP_106126621	--TATGL-L-A---I--S	PTT---------------------
Quadrisphaera granulorum	WP_109775565	--TAT-V-L-A---L-VS	-ATF--GR---V------------
Quadrisphaera sp. DSM 44207	WP_092863977	--TAT-V-L-A---L-VE	-ATF--GR---V------------
Raineyella antarctica	WP_092610267	-LTVRNL---A---L--E	PVSFQ-G---K-------------
Rothia sp. ND6WE1A	WP_068168439	--TVTNL-L-A---L-M-	EVNF-IDK--K-------------
Saccharomonospora azurea	WP_005437629	--TATGL-L-A-S-I--S	DTT---------------------
Saccharomonospora cyanea	WP_005455793	--SATGL-L-A-S-I--S	DTT---------------------
Saccharomonospora glauca	WP_005463784	--TATGL-L-A-S-I--S	DTT---------------------
Saccharomonospora halophila	WP_019812436	--TASGL-L-A-S-I--S	-AT---------------------
Saccharomonospora marina	WP_009154865	--TASGL-L-A-S-V--G	QTT---------------------
Saccharomonospora paurometabol	WP_007027016	--TASGL-L-A-S-I--S	-AT---------------------
Saccharomonospora saliphila	WP_019813444	--TASGL-L-AAS-I--S	DTT--I------------------
Saccharomonospora sp. CNQ490	WP_024874223	--TATGL-L-A-S-I--S	-TT---------------------
Saccharomonospora sp. CUA-673	WP_075850534	--TATGLQL-A-S-I---	DTT---------------------
Saccharomonospora sp. LRS4.154	WP_081190664	--TATGL-L-A-S-I--S	-TT---------------------
Saccharomonospora viridis	WP_015785978	--TATGL-L-A-S-I--S	DTT---------------------
Saccharomonospora xinjiangensi	WP_006236591	--TATGL-L-A-S-I--S	DTT---------------------
Saccharopolyspora antimicrobic	WP_093155521	--SATGL-L-A-S-I--S	DAM--------V------------
Saccharopolyspora erythraea	WP_009942936	--SATGL-L-A-S-I--S	EAT---------------------
Saccharopolyspora flava	WP_093417168	--SATGL-L-A-S-I--S	DAT---------------------
Saccharopolyspora rectivirgula	WP_029721879	--SATGL-L-A-S-I--S	DAT---------------------
Saccharopolyspora shandongensi	WP_093260267	--SATGL-L-A-S-I--S	DAT--I------------------
Saccharopolyspora sp. H219	RRO18098	--SATGL-L-A-S-I--S	DAT---------------------
Saccharopolyspora spinosa	WP_010310665	--SATGL-L-A-S-I--S	DAT---------------------
Saccharothrix australiensis	WP_121007902	--TAT-L-L-A-S-I--S	DAT---------------------
Saccharothrix carnea	WP_106618693	--TAT-L-L-A-S-I--S	DAT---------------------
Saccharothrix espanaensis	WP_015103571	--TAT-L-L-A-S-I--S	DAT---------------------
Saccharothrix sp. ALI-22-I	WP_077005248	--TAT-I-L-A-S-I--S	DAT---------------------
Saccharothrix sp. CB00851	WP_073900189	--TAT-L-L-A-S-I--S	DAT---------------------
Saccharothrix sp. NRRL B-16314	WP_033440333	--TAT-V-L-A-S-I--S	-AT----A----------------
Saccharothrix sp. NRRL B-16348	WP_053719180	--TAT-L-L-A-S-I--S	DAT----A----------------
Saccharothrix sp. ST-888	WP_045303958	--SA-AL-L-A---I-IE	SANF-IA-----------------
Saccharothrix syringae	WP_033433350	--TAT-L-L-A-S-I--S	-AT---------------------
Saccharothrix texasensis	WP_123746640	--TAT-L-L-A-S-I--S	DAT----S----------------
Saccharothrix variisporea	WP_121228097	--TAT-L-L-A-S-I--S	DAT---------------------
Salinispora arenicola	WP_029026749	--TATGL-L-A-S-I--S	DTT---------------------
Salinispora pacifica	WP_018216241	--TATGL-L-A-S-I--S	DTT---------------------
Salinispora sp. 13K206	WP_111137625	--TATGL-L-A---I--S	DTT---------------------
Salinispora tropica	WP_026275267	--TATGL-L-A-S-I--S	DTT---------------------
Sciscionella marina	WP_020497744	--SATEL-L-A-S-I-VS	-AN---------------------
Sciscionella sp. SE31	WP_031469945	--SATEL-L-A-S-I-VS	-AN---------------------

Other Bacteria (1/>1000)

Sinomonas humi	WP_043119763	--TVQ-L-L-A---L-M-	-VTF-IDK--K-------------
Sinomonas mesophila	WP_077488504	--TVS-L-L-A---L-M-	EVSF-IDK--K-------------
Sphaerisporangium album	WP_114030193	--IAS-I-L-A-S-L-IE	-ASF--N-----------------
Sphaerisporangium sp. 7K107	WP_111167389	--ISS-I-L-A---L--E	-ASF--N---K-------------
Sphaerisporangium sp. LHW63015	WP_113981775	--ISS-I-L-A---L--E	-ASF--N---K-------------
Spirillospora albida	WP_030167631	--IA--I-L-A-S-L-IE	AASF--N----V-F----------
Sporichthya polymorpha	WP_019877376	--TATGL-L-----L--G	AATF--G----V------------
Stackebrandtia nassauensis	WP_013017719	--SVTGL-L-A-S-I--S	DIN---------------------
Streptacidiphilus albus	WP_034090508	--AA-AL-L-A---I-IE	SATF--A-----------------
Streptacidiphilus carbonis	WP_042401895	--AA-AI-L-A---I--E	SASF--A-----------------
Streptacidiphilus jeojiense	WP_030266543	--AA-AI-L-A---I--E	SASF--A-----------------
Streptacidiphilus jiangxiensis	WP_042446285	---A--I-L-A---I-IE	SATF--A-----------------
Streptacidiphilus pinicola	WP_111501140	---A--I-L-A---I-IE	SATF--A-----------------
Streptacidiphilus rugosus	WP_037607250	--AA--I-L-A---I-IE	SASF--AA----------------
Streptoalloteichus hindustanus	WP_073480595	--TVT-L-L-A-S-I--S	DAT---------------------
Streptomonospora alba	WP_040269812	-LIAS-L-L---S-L--E	PTT---AA---V------------
Streptomyces afghaniensis 772	EPJ37983	--TVR-VD------L--S	-VSFH-S-----------------
Streptomyces albus	WP_030544575	--TATGL-L-A---L-IE	SASF-IAK----------------
Streptomyces alni	WP_093714375	--TATGI-L-A---V-IE	SASF-IAK----------------
Streptomyces armeniacus	AXK34206	--TASSL-L-A---V-IE	TATF--A----V------------
Streptomyces atratus	WP_037691628	--TASGI-L-A---I-IE	SANF-IAK----------------
Streptomyces azureus	GAP45962	--TVR-ID--A---L--S	-VSFH-S-----------------
Streptomyces catenulae	WP_030283071	--TASGL-L-A---V-I-	SASF-IAK---V------------
Streptomyces cattleya	WP_014141797	--TATGL-L-A---V-IE	SATF-IAK----------------
Streptomyces cavourensis	RBL80051	--TATGI-L-A---I-IE	SASF-IAK----------------
Streptomyces decoyicus	WP_030087498	--TASGL-L-A---I-IE	SASF-IAK----------------
Streptomyces erythrochromogene	WP_031149031	--TATGI-L-A---V-IE	SASF-IAK----------------
Streptomyces exfoliatus	WP_024756250	--TATGI-L-A---V--E	SASF--AK----------------
Streptomyces fragilis	WP_108953997	--TATGI-L-A---V-IE	SASF--AK----------------
Streptomyces gilvigriseus	WP_071655052	--TASKV-L-A---I-IE	SASF--G-----------------
Streptomyces globosus	WP_114057030	--TATGI-L-A---I-IE	SASF--AK----------------
Streptomyces griseocarneus	WP_121797657	--TATGI-L-A---V-IE	SASF--AK----------------
Streptomyces griseorubens	WP_037642385	--TAT-L-L-----V--Q	PSSF-IAK----------------
Streptomyces guanduensis	WP_093784720	--TATGI-L-A---V--E	SASF--AK----------------
Streptomyces hoynatensis	WP_120679226	--TATGI-L-A---I--E	SASF--AK---V------------
Streptomyces hundungensis	WP_120724605	--TATGI-L-A---V-IE	SASF--AK----------------
Streptomyces hyalinus	GCD99540	--NATNV-L-A---I--E	PASF--AA----------------
Streptomyces indicus	WP_093614561	--TATGI-L-A---I-IE	NATF--AK----------------
Streptomyces kanasensis	WP_058943571	--TATGL-L-A---V-IE	SASF-IAK----------------
Streptomyces lavendulae	WP_030233425	--TATGV-L-A---V-IE	SASF--AK----------------
Streptomyces megasporus	WP_031510526	--TATGL-L-A---V-IE	SASF-ITR----------------
Streptomyces nanshensis	WP_070017835	--SATGL-L-A---I-IE	SATF--AK----------------
Streptomyces niveus	WP_078077978	--TATGV-L-A---I-IE	SASF-IAK----------------
Streptomyces oceani	WP_070197710	--TASGL-L-----L-IE	SATF-IA-----------------
Streptomyces odonnellii	WP_046508696	--TATGV-L-A---V-IE	SASF--AK----------------
Streptomyces pathocidini	WP_055472014	--TATGL-L-A---I-IE	SASF-IAK---V------------
Streptomyces paucisporeus	WP_073496991	--TATGL-L-A---V-IE	SASF--AK----------------
Streptomyces pharetrae	WP_086172530	--TAT-L-L-----V--Q	PSSF-IAK----------------
Streptomyces phytohabitans	WP_121516223	--TASRL-L-A---V-IE	TADF-IAA---V------------
Streptomyces platensis	WP_085927250	--TASGL-L-A---I-IE	SASF-IAK----------------
Streptomyces puniciscabiei	AOR30117	--TAR-V-L-A---L--S	-ISCT-S-----------------
Streptomyces qinglanensis	WP_074998057	--TATGL-L-A---V-IE	SATF-IAK----------------
Streptomyces reticuli	CUW25897	--TVR-A-L-A---L--S	-VSFT-S-----------------
Streptomyces roseochromogenus	EST36200	--IAR-V-L-A---L--S	-TSFT-S-----------------
Streptomyces roseus	WP_048476733	--TATGV-L-A---I-IE	SASF-IAK----------------
Streptomyces rubidus	WP_069465928	--TATGL-L-A---V-IE	TASF--AK----------------
Streptomyces scabrisporus	WP_020553539	--NATNV-L-A---I--E	PASF--AA----------------
Streptomyces somaliensis	WP_010468920	--TATGL-L-A---V-IE	SASF-IAK----------------
Streptomyces sp. 150FB	WP_040020469	--TATGV-L-A---I-IA	SASF-IAK----------------
Streptomyces sp. 3211.1	WP_119096402	--TATGI-L-A---V-IE	SASF-IAK----------------
Streptomyces sp. 4121.5	WP_100840155	--SA-AL-L-A---I-IE	SASF-IA-----------------
Streptomyces sp. 4F	WP_058917225	--TAT-L-L-----V--Q	PSSF-IAK----------------
Streptomyces sp. AA4	EFL06659	L-TATGL-L-A-S-I--N	-AT--I-S----------------
Streptomyces sp. AC230	WP_114622333	--TATGV-L-A---I-IE	SASF-IAK----------------
Streptomyces sp. Ag82_G6-1	SOD49635	--TVR-VD------L--S	-VSFH-S-----------------
Streptomyces sp. Amel2xB2	WP_111603127	--TATGL-L-A---I-IE	SATF--AK----------------
Streptomyces sp. AmelKG-D3	WP_099219262	--TATGI-L-A---V-IE	SASF-IAK----------------
Streptomyces sp. C	EFL14436	--TATGI-L-A---I-IE	NASF--AK----------------
Streptomyces sp. CB00072	WP_073867443	--TATGI-L-A---I-IE	SASF-IAK----------------
Streptomyces sp. CB00455	WP_073913899	--TATGV-L-A---V-IE	SASF--AK----------------
Streptomyces sp. CB01201	WP_100574354	--TATGI-L-A---V-IE	SASF--AK----------------
Streptomyces sp. CB03911	WP_073928788	--SA-AL-L-A---V-IE	SASF-IA-----------------
Streptomyces sp. CC53	WP_071281234	--TATGL-L-A---V-IE	KASF-IAR----------------
Streptomyces sp. CMB-StM0423	WP_101425225	--TATGL-L-A---I--E	SATF-IAK----------------

Other Bacteria (1/>1000)

Streptomyces sp. CNH099	WP_027753498	--TATGL-L-A---V--E	SATF-IAR---------------
Streptomyces sp. CNQ329	WP_027772978	--TATGL-L-A---I--E	SATF--AR---------------
Streptomyces sp. CNS606	WP_027762366	--TASRL-L-A---I-I-	SASF-IAK---------------
Streptomyces sp. CNT360	WP_027736543	--TATGL-L-A---I-IE	SATF-IAK---------------
Streptomyces sp. CNT371	WP_027743007	--TATGL-L-A---I--E	SATF--AK---------------
Streptomyces sp. CT34	WP_043263791	--TASGL-L-A---I-IE	SASF--AK---------------
Streptomyces sp. DJ	PLW67095	--TATGI-L-A---V-IE	SASF-IAK---------------
Streptomyces sp. DvalAA-14	WP_093737621	--TATGI-L-A---I-IE	SASF--AK---------------
Streptomyces sp. DvalAA-19	WP_093750487	--TATGI-L-A---I-IE	SASF-IAK---------------
Streptomyces sp. DvalAA-43	WP_093539741	--TASGI-L-A---I-IE	SASF--AK---------------
Streptomyces sp. GP55	WP_101382831	--SA-TL-L-A---I-IE	SASF--A----------------
Streptomyces sp. ICBB 8177	WP_109451081	--TATGL-L-A---V--E	SASF-IAK---------------
Streptomyces sp. LHW50302	WP_114016971	--TATGL-L-A---I-IE	SATF--AK---------------
Streptomyces sp. LcepLS	WP_093582009	--TATGI-L-A---I-IE	NASF--AK---------------
Streptomyces sp. MJM1172	WP_073780606	--TATGI-L-A---V-IE	SASF--AK---------------
Streptomyces sp. MP131-18	WP_077062175	--TATGI-L-A---I--E	STSF-IAQ---------------
Streptomyces sp. NBRC 110027	WP_042161602	--TASGL-L-A---I-IE	SASF-IAK---------------
Streptomyces sp. NEAU-YY421	RFU88455	--TASNI-L-A---I-IE	NASF-IAK---------------
Streptomyces sp. NL15-2K	WP_124443996	--TAT-L-L-----V--Q	PSSF-IAK------I---------
Streptomyces sp. NRRL B-24484	WP_030266745	--SA-AL-L-A---V-IE	SASF-IA----------------
Streptomyces sp. NRRL B-24572	WP_086830175	--TASGI-L-A---I-IE	SASF--AK---------------
Streptomyces sp. NRRL F-2580	WP_030716919	--TATGV-L-A---I-IE	SASF--AK---------------
Streptomyces sp. NRRL F-2664	WP_030766833	--TATGI-L-A---V-IE	SASF-IAK---------------
Streptomyces sp. NRRL F-4474	WP_030849666	--TATGI-L-A---V-IE	SASF-IAK---------------
Streptomyces sp. NRRL F-5630	WP_030996298	--TATGI-L-A---I-IE	NASF--AK---------------
Streptomyces sp. NRRL F-5727	WP_031002896	--TASGI-L-A---I-IE	NASF--AK---------------
Streptomyces sp. NRRL F-6131	WP_030305767	--SA-GL-L-A---I-IE	SASFK-A----------------
Streptomyces sp. NRRL S-1448	WP_030416606	--TATGL-L-A---I-IE	SASF--AR---------------
Streptomyces sp. NRRL S-1813	WP_030988396	--TASGL-L-A---I-IE	SASF-IAK---------------
Streptomyces sp. NRRL S-384	WP_030911088	--SA-AL-L-A---I-IE	SASF-IA----------------
Streptomyces sp. NRRL S-455	WP_030249965	--TVR-VD------L--S	-VSFH-S----------------
Streptomyces sp. NRRL S-474	WP_030896371	--TVR-VD------L--S	-VSFH-S----------------
Streptomyces sp. NRRL S-481	WP_030951469	--TVR-VD------L--S	-VSFH-S----------------
Streptomyces sp. NRRL S-495	KJY35471	--SV-GL-L-A---I-IE	SANFK-AS---------------
Streptomyces sp. NRRL S-87	WP_030203201	--TATGI-L-A---I-IE	NASF--AK---------------
Streptomyces sp. PCS3-D2	WP_037930202	--TATGI-L-A---V-IE	SASF-IAK---------------
Streptomyces sp. PsTaAH-137	WP_111667014	--TASGI-L-A---I-IE	NANF--AK---------------
Streptomyces sp. RSD-27	KIF01654	--TATGI-L-A---V-IE	SASF-IAK---------------
Streptomyces sp. Root431	WP_056646184	--TATGI-L-A---V-IE	SASF--AK---------------
Streptomyces sp. Ru87	WP_098751261	--TATGL-L-A---L-IE	SASF--AK---------------
Streptomyces sp. SDr-06	WP_114035939	--TATGI-L-A---V-IE	SASF--AK---------------
Streptomyces sp. SPB074	WP_008748663	--TATGI-L-A---I-IE	NASF--TK---------------
Streptomyces sp. SPB78	WP_009064025	--TATGI-L-A---I-IE	NASF--AK---------------
Streptomyces sp. TAA204	WP_028431441	--TASGL-L-A---I-IE	SATF--AK---------------
Streptomyces sp. TAA486	WP_028435538	--TATGL-L-A---I-IE	SATF-IAR---------------
Streptomyces sp. TLI_146	WP_101387699	--TATGI-L-A---I-IE	NASF--AK---------------
Streptomyces sp. TLI_235	WP_095872667	--SA-AL-L-A---V-IE	SASF-IA----------------
Streptomyces sp. TSRI0281	WP_073720209	--TASGI-L-A---I-IE	SASF--AK---------------
Streptomyces sp. Tu 6176	EYT82136	--TVR-A-L-A---L--S	-ISFT-S----------------
Streptomyces sp. TverLS-915	WP_093816448	--TATGI-L-A---I-IE	NASF--AK---------------
Streptomyces sp. URHA0041	WP_033172269	--TATGL-L-A---I-IE	SASF--AR---------------
Streptomyces sp. WM4235	WP_053678585	--TATGV-L-A---I-IE	SASF--AK---------------
Streptomyces sp. WM6372	WP_053692586	--TATGI-L-A---V-IE	SASF-IAK---------------
Streptomyces sp. WM6378	WP_053724054	--TATGI-L-A---V-IE	SASF--AK---------------
Streptomyces sp. WMMB 322	WP_055485343	--TATGL-L-A---I-IE	SATF-IAR---------------
Streptomyces sp. WMMB 714	WP_045864327	--TATGL-L-A---I-IE	SATF--AK---------------
Streptomyces sp. WZ.A104	WP_096628172	--TATGI-L-A---I-IE	SASF-IAK---------------
Streptomyces sp. YIM 130001	WP_119289816	--TASNI-L-A---I-IE	NASF--AK---------------
Streptomyces specialis	WP_059009464	--TATGI-L-A---I-IE	SASF-IAT---------------
Streptomyces subrutilus	WP_069919723	--TATGI-L-A---I--E	SASF-IAK---------------
Streptomyces tateyamensis	WP_110673464	--SA-AL-L-A---I-IE	TASF-IA----------------
Streptomyces thermoautotrophic	WP_066886066	--TAL-I-L-A-S-I--E	NASF--A----V-----------
Streptomyces thermoautotrophic	WP_067069418	--TAL-I-L-A-S-I--E	NASF--A----V-----------
Streptomyces thermolilacinus	WP_023589330	--TATGL-L-A---V-IE	SASF-IAR---------------
Streptomyces tsukubensis	WP_077971976	--TATGI-L-A---I-IE	SANF--AK---------------
Streptomyces venezuelae	WP_055644246	--TATGI-L-A---V-IE	SASF--AK---------------
Streptomyces vietnamensis	WP_041128586	--TASGI-L-A---I-IE	NASF--AK---------------
Streptomyces xanthophaeus	WP_031148011	--TATGI-L-A---V-IE	SASF--AK---------------
Streptomyces xinghaiensis	WP_019709628	--TATGL-L-A---L-IE	SASF-IAK---------------
Streptomyces yanglinensis	WP_103884078	--TATGI-L-A---I-IE	SASF--AK---------------
Streptomyces yeochonensis	WP_037905892	--TATGV-L-A---V-IE	SASF--AK---------------
Streptosporangiaceae bacterium	WP_119925057	--IAQ-I-L-A-S-L-IE	-ASF--N----------------
Streptosporangium amethystogen	WP_030904787	---AS-I-L-A---L-IE	-ASF--N---K------------
Streptosporangium minutum	WP_086575125	---AS-I-L-A---L-IE	-ASF--N---K------------

Other Bacteria (1/>1000)

Streptosporangium roseum	WP_012892442	---AS-I-L-A---L-IE	-ASF--N---K-------------
Streptosporangium subroseum	WP_089210265	---AS-I-L-A---L-IE	NASF--N---K-------------
Tessaracoccus aquimaris	WP_077685673	-LQVK-I---A---L--S	PVSFQ-I-----------------
Tessaracoccus oleiagri	WP_093252025	-LQAR-I---A---L--S	PISFQ-I-----------------
Tessaracoccus sp. Marseille-P5	WP_108871696	-LRA--V---A---L--S	PVSFQII-----------------
Tetrasphaera australiensis	WP_048696442	--AAS-L-L---S-L---	EATF-IN----V------------
Tetrasphaera duodecadis	WP_101394014	--TAAAV-L-A-S-L---	-ATF--A-----------------
Tetrasphaera sp. Soil756	WP_055812460	--TAAAV-L-A-S-L--E	-ATF--A-----------------
Thermasporomyces composti	WP_115848846	--TASNL-L-A---L--A	NASF--S---K-------------
Thermoactinospora rubra	WP_084958256	--IAT-I-L-A-S-L-IE	NATF--N---K-------------
Thermobifida cellulosilytica	WP_068754944	-LIAS-L-L-----L--E	PTSFQ-G-----------------
Thermobifida fusca	WP_011292304	--IAS-L-L-----L--A	PASFQ-G-----------------
Thermobifida halotolerans	WP_068693927	-LIAS-L-L-----L--E	PTSFQ-G-----------------
Thermocrispum agreste	WP_028846109	--TATGL-L-A-S----A	DTT---------------------
Thermocrispum municipale	WP_028849308	--TATGL-L-A-S----A	ETT---------------------
Thermomonospora curvata	WP_012852588	--TAT-I-L-A---L-IE	KATF--N----V------------
Thermostaphylospora chromogena	WP_093261755	--IASEI-L-A---L---	-ASF--N---K-------------
Umezawaea tangerina	WP_106191475	--TATNI-L-A-S-I--S	EAN---------------------
Verrucosispora maris	WP_013734925	--TATGL-L-A---I--S	DTT---------------------
Verrucosispora sediminis	WP_093401430	--TATGL-L-A---I--S	DTT---------------------
Verrucosispora sp. CNZ293	WP_099844850	--TATGL-L-A---I--S	DTT---------------------
Verrucosispora sp. LHW63014	WP_117227193	--TASGL-L-A---I--S	DTT---------------------
Verrucosispora sp. ts21	WP_102658502	--TATGL-L-A---I--S	DTT---------------------
Xiangella phaseoli	WP_092382498	--TASGL-L-A---I--S	DTT---------------------
Xylanimonas cellulosilytica	WP_012878184	--TAHAV-L-I---V--T	PSTF-IA-----------------
Yuhushiella deserti	WP_092533330	--TATGL-L-A-S-V--A	DTD---------------------
Zhihengliuella halotolerans	WP_102157513	--SVA-L-L-A---L-ME	-VSF-IDS--K-------------
